# Characterization of Bacterial, Archaeal and Eukaryote Symbionts from Antarctic Sponges Reveals a High Diversity at a Three-Domain Level and a Particular Signature for This Ecosystem

**DOI:** 10.1371/journal.pone.0138837

**Published:** 2015-09-30

**Authors:** Susana Rodríguez-Marconi, Rodrigo De la Iglesia, Beatriz Díez, Cássio A. Fonseca, Eduardo Hajdu, Nicole Trefault

**Affiliations:** 1 Center for Genomics and Bioinformatics, Faculty of Sciences, Universidad Mayor, Camino La Pirámide 5750, Santiago, Chile; 2 Department of Molecular Genetics and Microbiology, Pontificia Universidad Católica de Chile, Alameda 340, Santiago, Chile; 3 Museu Nacional, Universidade Federal do Rio de Janeiro, Quinta da Boa Vista s/n, 20940–040, Rio de Janeiro, Rio de Janeiro, Brazil; Australian Institute of Marine Science, AUSTRALIA

## Abstract

Sponge-associated microbial communities include members from the three domains of life. In the case of bacteria, they are diverse, host specific and different from the surrounding seawater. However, little is known about the diversity and specificity of Eukarya and Archaea living in association with marine sponges. This knowledge gap is even greater regarding sponges from regions other than temperate and tropical environments. In Antarctica, marine sponges are abundant and important members of the benthos, structuring the Antarctic marine ecosystem. In this study, we used high throughput ribosomal gene sequencing to investigate the three-domain diversity and community composition from eight different Antarctic sponges. Taxonomic identification reveals that they belong to families Acarnidae, Chalinidae, Hymedesmiidae, Hymeniacidonidae, Leucettidae, Microcionidae, and Myxillidae. Our study indicates that there are different diversity and similarity patterns between bacterial/archaeal and eukaryote microbial symbionts from these Antarctic marine sponges, indicating inherent differences in how organisms from different domains establish symbiotic relationships. In general, when considering diversity indices and number of phyla detected, sponge-associated communities are more diverse than the planktonic communities. We conclude that three-domain microbial communities from Antarctic sponges are different from surrounding planktonic communities, expanding previous observations for Bacteria and including the Antarctic environment. Furthermore, we reveal differences in the composition of the sponge associated bacterial assemblages between Antarctic and tropical-temperate environments and the presence of a highly complex microbial eukaryote community, suggesting a particular signature for Antarctic sponges, different to that reported from other ecosystems.

## Introduction

Symbiosis, a permanent or long-lasting association between two or more different species of organisms, has played a key role in the generation of biological diversity [1,2]. Symbiotic interactions involving microorganisms are essential to the marine environment ecology, and sponges are a remarkable exponent of this kind of interaction. These sessile, filter-feeding metazoans harbor diverse microbial communities from the three domains of life [3], that accounts for up to 40% of total sponge biomass [3,4]. Interactions between sponges and microorganisms range from mutualism to commensalism and parasitism, and show an impact upon host defense, nutrition and metabolism [5].

Sponge-associated bacterial communities have been widely studied, and some general conclusions have arisen: (i) they are host specific, (ii) they are different from planktonic communities in the surrounding water and (iii) despite being phylogenetically different, they share functional characteristics that allow them to live in symbiosis [6–9]. Temperate and tropical water studies indicate the presence of around 40 bacterial phyla or candidate phyla, in which Proteobacteria, Actinobacteria, Chloroflexi, Bacteroidetes and Cyanobacteria often predominate [9–13]. Over 100 sponge-specific clusters like candidate phylum Poribacteria [14] and *Candidatus* Synechococcus spongiarum [15] are proposed, although a third of them have been recently found in the surrounding seawater, but at very low concentrations [16]. Culture dependent and molecular studies, including high throughput sequencing of ribosomal genes, also demonstrate that marine sponges are habitats for highly diverse symbionts from the Archaea and Eukarya domains of life. In the Archaea domain, two major phyla are described, with Thaumarchaeota considerably dominating sponge-associated microbial communities in several sponges from Arctic and Irish deep-sea environments [17,18]. Regarding eukaryotic symbionts, 11 phyla belonging to six supergroups of fungi and protists have been described to inhabit sponge tissues [19–22]. However, the majority of these studies focus on the description of one of the microbial components (i.e. bacteria, archaea, fungi and protist) of the sponge microbiome, and descriptions of whole sponge-associated microbial communities are scarce.

Marine sponges are classified in two groups according to the abundance of bacterial symbionts, High Microbial Abundance (HMA) and Low Microbial Abundance (LMA). HMA sponges host a diverse array of bacterial communities, including an important photosynthetic fraction, i.e. cyanobacteria. In contrast, LMA sponges harbor less diverse and abundant bacterial communities, highly dominated by Proteobacteria and Bacteroidetes [23]. There is yet no evidence for a similar dichotomy for the eukaryote or archaeal components of the sponge microbiome.

While the majority of studies about sponge-associated microbial communities have been developed in temperate and tropical environments, there is limited information about other habitats, such as polar areas and the deep sea. Sponges can occupy up to 80% of available surfaces in the Antarctic benthos, and play a key role in community dynamics and structure [24]. In this geographically isolated ecosystem, with harsh environmental conditions, such as continual near-freezing temperatures and cyclical sea-ice formation [25], Antarctic marine sponges constitute a particularly attractive model for the study of symbiosis. In 2004, Webster et al. in a pioneer study, explored the microbial communities of five species of Antarctic sponges using clone libraries and denaturing gradient gel electrophoresis (DGGE). The authors indicated that a significant proportion of the retrieved diversity was sponge specific, with representatives of Gamma and Alpha Proteobacteria and Bacteroidetes, diatoms, dinoflagellates, and an uncultured Crenarchaeota representative [20]. Since then, there have been several approximations to the description of Antarctic microbial communities associated to sponges, but primarily focused on cultivable microorganisms or microscopic observations [19,26,27].

Our study aims to describe and compare Antarctic sponge microbial communities from the three-domains. The following questions were addressed: 1) How is the diversity and community composition of microorganisms at the three-domain level associated to Antarctic marine sponges, compared to the planktonic communities in the surrounding water? 2) Are bacterial, archaeal and eukaryote Antarctic sponge-microbial communities host-specific and is there a core-microbiome associated to them? 3) Are these communities similar to the ones described in sponges from other habitats? If there are differences, what are the key components supporting these differences? To this end, we comprehensively describe and compare the microbial diversity of Bacterial, Archaeal and Eukaryote microorganisms associated to eight different Antarctic sponges collected in Fildes Bay (King George Island, South Shetlands), and the surrounding seawater, using tag sequencing of hypervariable regions from 16S and 18S rRNA genes. This study provides new insights into the characterization of whole microbial communities in sponges in general, and of the microbial communities of Antarctic sponges in particular.

## Methodology

### Sample collection

The Instituto Antártico Chileno-INACH issued the permission for sampling. Sponge samples (N = 8) were collected by scuba diving at 5, 17, 20 and 27m, on January 2013 at two sites in Fildes Bay (King George Island, South Shetlands, Antarctica ‒ Site1: 62°11´59.1´´S, 58°56´35.1´´ W, Site 2: 62°11´17.7´´S, 58°52´22.8´´W). Sampling sites were 3.8 km distant from each other. Sponges were kept individually in plastic bags containing natural seawater at 4°C until processing within a few hours after collection. One seawater sample (SW) was collected at 27 m depth using a 5 L Niskin bottle, approximately 5 meters away from sponge location at Site 2. This sample was prefiltered on board through 150 μm pore mesh to remove large particles, stored in an acid-washed carboy and kept in the dark until processing in the lab. Salinity, fluorescence, oxygen and temperature data were obtained using a SBE 911 plus (SeaBird) CTD profiler.

### Sponge treatment

Each sponge individual was rinsed 3 times with sterilized seawater, carefully cleaned under stereomicroscope to remove dirt and ectoparasites and stored at -80°C until processing. From each sponge, triplicate tissue samples of ~1 cm^2^ were extracted with a sterile scalpel blade.

To separate the microbial community intimately associated to the sponge, from that loosely attached (due to filter feeding or incidental association), we followed a protocol adapted from Thomas et al. (2010) [28], that consisted in washing the sponge material, disruption of tissue, filtration and centrifugation. The tissue was rinsed three times with artificial sterile seawater (ASW, 25 g L^-1^ NaCl, 0,8 g L^-1^ KCl, 1 g L^-1^ Na_2_SO_4_, 0,04 g L^-1^ NaHCO_3_) and cut into small pieces (~1 mm^2^). The fragments were mixed with 500 μL of ASW and processed in a TissueLyser II (Qiagen) with a 5 mm steel bead in a 2 mL tube for 3 minutes at 30 hz. Sponge pulp was suspended in 10 mL of ASW and filtered through a 115 μm-pore-size mesh. Sponge cells were separated by centrifugation at 100 g for 15 min at 4°C and the supernatant was centrifuged again for 20 min at 8,800 g. The obtained pellets containing the associated microbial community were verified by epifluorescence microscopy.

### Seawater treatment

For surrounding planktonic community analysis, seawater sample was filtered through 20 (NY20), 3 (GSWP0) and 0.2 μm (GPWP) pore size filters 47 mm in diameter (Millipore), using a Swinnex holder system and a Cole Parmer 1–600 rpm peristaltic pump. Filters were stored in 2 mL cryovials at -20°C until DNA extraction.

### Sponge identification

Ethanol fixed sub-samples from each sponge were used for taxonomic identification following standard protocols for the obtention of dissociated spicules and thick sections, as described in detail by Hajdu et al. (2011) [29]. Identification was done after comparisons with the specialized literature, as well as with reference biological samples deposited in the MNRJ collection.

Voucher fragments of sponge specimens were deposited in the Porifera collection of Museu Nacional/UFRJ, under accession numbers MNRJ18648 A to K.

### DNA extractions

Genomic DNA from the pellet obtained after sponge treatment was extracted with the PowerSoil DNA Isolation Kit (MOBIO).

For seawater planktonic communities, filters were thawed and half of them were cut into small pieces, while the other half was kept at -20°C as backup. Each sample was incubated in lysis buffer (TE1x/NaCl 0.15 M), with 10% SDS and 20 mg mL^-1^ proteinase K and incubated at 37°C for 1 hour. DNA was extracted using 5 M NaCl and N-cetyl N,N,Ntrimethylammonium bromide (CTAB) extraction buffer (10% CTAB, 0.7% NaCl), incubated at 65°C for 10 min. Protein removal was done using a conventional phenol-chloroform method [30]. DNA was precipitated using isopropanol at –20°C for 1 h and resuspended in 50 μL milliq water after two ethanol 70% wash steps.

DNA integrity for both sponge-associated and surrounding planktonic microbial communities was evaluated by 0.8% agarose gel electrophoresis, quantified using a Quantifluor (Promega) with Quant-iT Picogreen (Invitrogen), and stored at -20°C until further analysis.

### Tag sequencing

Tag sequencing was performed for deep parallel taxonomic characterization of sponge-associated microbial communities and surrounding planktonic communities. The hypervariable regions V4 and V9 of 16S and 18S rRNA genes, respectively, were amplified for further sequencing. Amplification of V4 region was performed using 515Fseq and 806rcbc primer pairs [31] and V9 region using the three-domain primer 1391f and the eukaryal specific EukBr primer [32]. From each sponge DNA extraction replicate, an independent PCR amplicon was generated. In the case of DNA extractions from the seawater sample, each size fraction was amplified in triplicate. PCR reactions were performed in 35 μL final volume with Taq buffer 1x final concentration, 2 nM of MgCl_2_, 0.3nM of dNTPs, 0.3 μM of each primer, 2.5 units of GoTaq Flexi DNA Polymerase (Fermelo) and 1 to 5 ng of template DNA. Amplification conditions were 3 min of initial denaturation at 94°C, 28 cycles of 94°C for 30 s, 57°C (V4 16S rRNA) or 60°C (V9 18S rRNA) for 1 min and 72°C for 1.5 min, followed by a final extension of 72°C for 10 min. Illumina primer constructs were obtained from Earth Microbiome Project [33]. The three amplicons generated from the same sponge were pooled, as well as the three replicate amplicons from each size fraction from the seawater. Combined amplicons were quantified using a standard qPCR assay using a Library Quant Kit Illumina (Kapa) according to manufacturer instructions, equimolarly pooled and sequenced using Illumina Miseq following Caporaso et al. 2011 [31] protocol. Samples were sequenced in two runs for V4 16S rRNA and three runs for V9 18S rRNA. 11.5±0.5 pM of qPCR quantified amplicons pool was sequenced each time using a 300 cycles Illumina Miseq kit.

Raw sequence data were deposited in SRA under BioProject number PRJNA287634.

### Data analysis

Sequences were analyzed using Mothur software [34]. Initial reads were demultiplexed and assembled using *make*.*contigs*. Sequences from the different size fractions from the seawater sample were combined due to a high variability among sequence numbers in these samples, so analyses were conducted with only one seawater dataset. Primers were removed using Cutadapt [35]. Sequences less than 200 bp (16S rRNA) or 130 bp (18S rRNA) long, with ambiguous nucleotides and homopolymers longer than 8 pb were removed from further analysis. Alignment was computed using the recreated Silva SEED v119 [36] as reference. Chimeras were screened and removed using UCHIME [37]. For the 16S rRNA gene, after a first assignment against Silva v119 [36], chloroplast and mitochondria sequences were removed. In the case of 18S rRNA gene, Metazoa sequences were removed after a first assignment against the PR2 database [38]. OTUs at 97% similarity were generated using default settings and clustering was performed with furthest neighbor algorithm. The taxonomic assignations were performed against Silva v119 [36] for 16S rRNA gene and PR2 database [38] for 18S rRNA gene. OTUs formed by 10 or less sequences in the case of 16S rRNA gene and 5 or less sequences in the case of 18S rRNA gene, were removed from further analysis. Rarefaction curves and diversity indexes were obtained with Mothur. Beta-diversity analyses were computed using weighted Unifrac distances [39] in Mothur. Heatmaps were constructed using Heatplus and Gplots packages in R environment using square root transformed data of relative abundance of the 50 more abundant OTUs. Hierarchical clustering was generated with group average method.

To evaluate shared, variable, host-specific and core sponge microbiome, a threshold of presence for each microbial OTU was set in 0.1% (present if 0.1% or higher). OTUs shared by at least 6 sponges and absent in seawater were dessignated as core microbiome, OTUs shared by 2 to 5 sponges and absent in seawater were called variable community and OTUs present in only one sponge and absent in seawater were called host-specific community.

To compare bacterial microbiota between Antarctic sponges and the ones from other environments, data from the following articles were considered: Webster et al. (2010) [6], Lee et al. (2011) [9], Jackson et al. (2012) [40], Cleary et al. (2013) [41] and Easson & Thacker (2014) [42]. These articles were selected because data presented were obtained from high throughput sequencing, they cover a wide range of environments, and the relative abundance of bacterial phyla in each sponge analyzed was easily accessible. Relative abundances of bacterial phyla were used to calculate pairwise similarities among samples using the Bray–Curtis similarity coefficient [43]. Bray–Curtis similarity matrices were visualized using hierarchical cluster, and Analysis of Similarity (ANOSIM) was calculated to test the significance of differences among different environments (temperate, polar, cold and tropical), using PRIMER v6 for Windows (PRIMER-E Ltd, Plymouth, UK).

Figures were generated with Graphpad Prism 6 and R version 3.1.2.

## Results

### Sponge taxonomic identification and environmental data

Eight sponges were collected from two sites at Fildes Bay (King George Island, South Shetlands Islands, Antarctica) during January 2013. Taxonomic identification of the sponges reveals that they belong to families Acarnidae, Chalinidae, Hymedesmiidae, Hymeniacidonidae, Leucettidae, Microcionidae, and Myxillidae (Table 1). For one of the sponges, identification was only possible at class level, due to problems during material shipment.

**Table 1:** Site and sponge species collected from different locations within Fildes Bay, King George Island, Antarctica.

Site	Depth (m)	Sponge species	Sample ID	Taxonomy		
				Class	Order	Family
S1	5	*Myxilla (Burtonanchora)* sp.	E4	Demospongiae	Poecilosclerida	Myxillidae
S1	20	*Clathria* sp.	E6	Demospongiae	Poecilosclerida	Microcionidae
S1	20	-	E7	Demospongiae	-	-
S1	17	*Kirkpatrickia variolosa*	E8	Demospongiae	Poecilosclerida	Hymedesmiidae
S2	27	*Hymeniacidon torquata*	E9	Demospongiae	Halichondrida	Hymeniacidonidae
S2	27	*Leucetta antarctica*	E10	Calcarea	Clathrinida	Leucettidae
S2	27	*Haliclona (Gellius)* sp.	E11	Demospongiae	Haplosclerida	Chalinidae
S2	27	*Megaciella annectens*	E12	Demospongiae	Poecilosclerida	Acarnidae

Physical parameters of the water column where almost equal between sampling sites. Temperature ranged from 0.74 to 1°C, salinity was 34 PSU and dissolved oxygen was 7.9 mL L^-1^ for both sites. Fluorescence ranged from 0.39 to 1.49 mg m^-3^, increasing with depth but without difference among sites (S1 Table).

### Diversity of microbial communities associated to Antarctic sponges

After all filtering steps, 16S rRNA (Bacteria/Archaea) and 18S rRNA (Eukarya) genes sequencing originated 2,057,252 and 2,288,470 sequences, respectively. The total number of OTUs, defined at a 97% similarity, was 5,120 for Bacteria/Archaea and 2,855 for Eukarya. Table 2 shows the sequence numbers summary for each sponge and dataset. Chao1 based rarefaction curves show a high coverage for all bacterial /archaeal samples and for five eukaryote samples (*Myxilla (Burtonanchora)* sp., *Clathria* sp., *K*. *variolosa*, *Haliclona (Gellius)* sp., *M*. *annectens* and SW) (S1 Fig).

**Table 2:** Summary of the sequencing data obtained.

	Bacteria/Archaea			Eukarya		
Sample ID	N° of initial sequences	N° of final sequences^1^	N° of OTUs	N° of initial sequences	N° of final sequences^1^	N° of OTUs
E4	2,572,059	128,300	1,278	1,037,567	907	110
E6	893,061	53,038	1,192	846,649	206,058	1,398
E7	211,168	155,680	1,277	1,586,807	40,310	283
E8	459,383	366,630	2,830	629,452	49,557	389
E9	456,746	378,598	1,723	1,045,025	2,310	166
E10	478,030	405,765	1,173	124,5326	29,736	522
E11	2,725,172	145,079	2,350	1,373,100	35,252	647
E12	1,093,026	68,118	989	1,817,084	286,916	1,313
SW	598,289	244,766	1,581	2,025,682	1,599,680	1,600

^1^ Represent high quality sequences without chimera, undesired (metazoan, chloroplast, mitochondria) and rare sequences.

**Fig 1:**
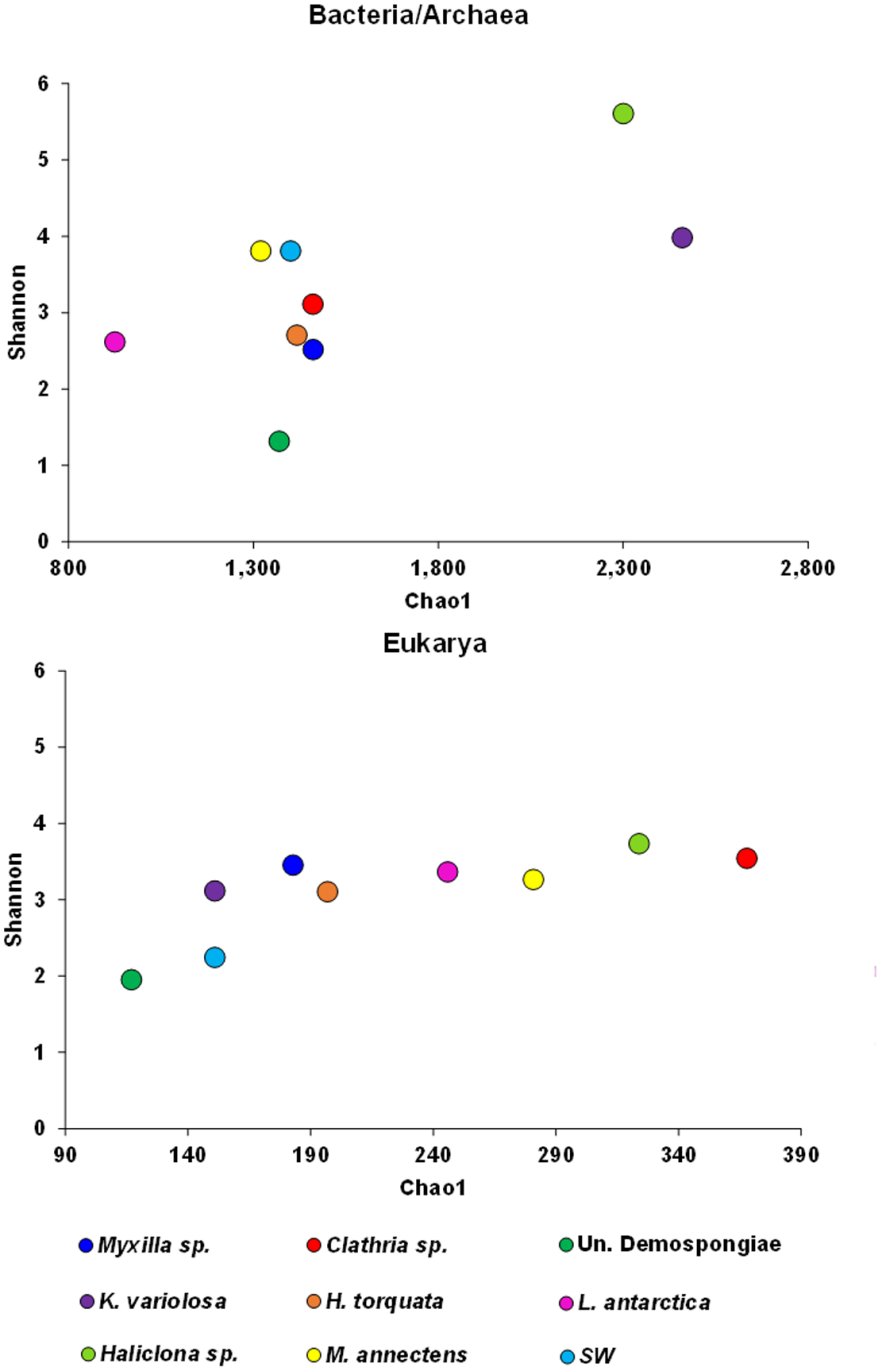
Diversity measures. Non-parametric Shannon and Chao1 estimator calculated with sequences rarefied to the sample with minimum sequence number (*Clathria* sp. with 53,038 and *Myxilla (Burtonanchora)* sp. with 907 sequences for Bacteria/Archaea and Eukarya, respectively). SW: surrounding seawater sample.

Ecological indices showed that, in general, bacterial/archaeal communities are richer and more diverse than eukaryote communities and that diversity patterns from sponge-associated microbiomes do not follow the same trend when comparing the two dataset (Fig 1). In the case of sponge associated bacterial/archaeal communities, mean and s.d. Observed richness (Sobs) was 1,195 ± 501, Expected richness (Chao1) was 1,590 ± 519, non-parametric Shannon (H’) was 3.1 ± 1.25 and Simpson (D) was 0.23 ± 0.21 (S2 Table). For the surrounding planktonic community, Sobs, Chao1 and D values were lower than the mean calculated for sponges. In contrast, H’ index was higher than the mean calculated for the eight sponges, with only two of them (i.e. *K*. *variolosa* and *Haliclona (Gellius)* sp.) displaying higher H’ values. In the case of microbial eukaryotes, surrounding planktonic community show lower Sobs, Chao1, H’ and D values than most sponge microbiomes (the only exception was E7) (S2 Table). Taken together, these results indicate that Bacteria/Archaea associated communities were less diverse than the surrounding seawater communities, while in the case of Eukarya associated communities, these were more diverse than their planktonic counterparts.

### Composition of the microbial community associated to Antarctic sponges

**Fig 2:**
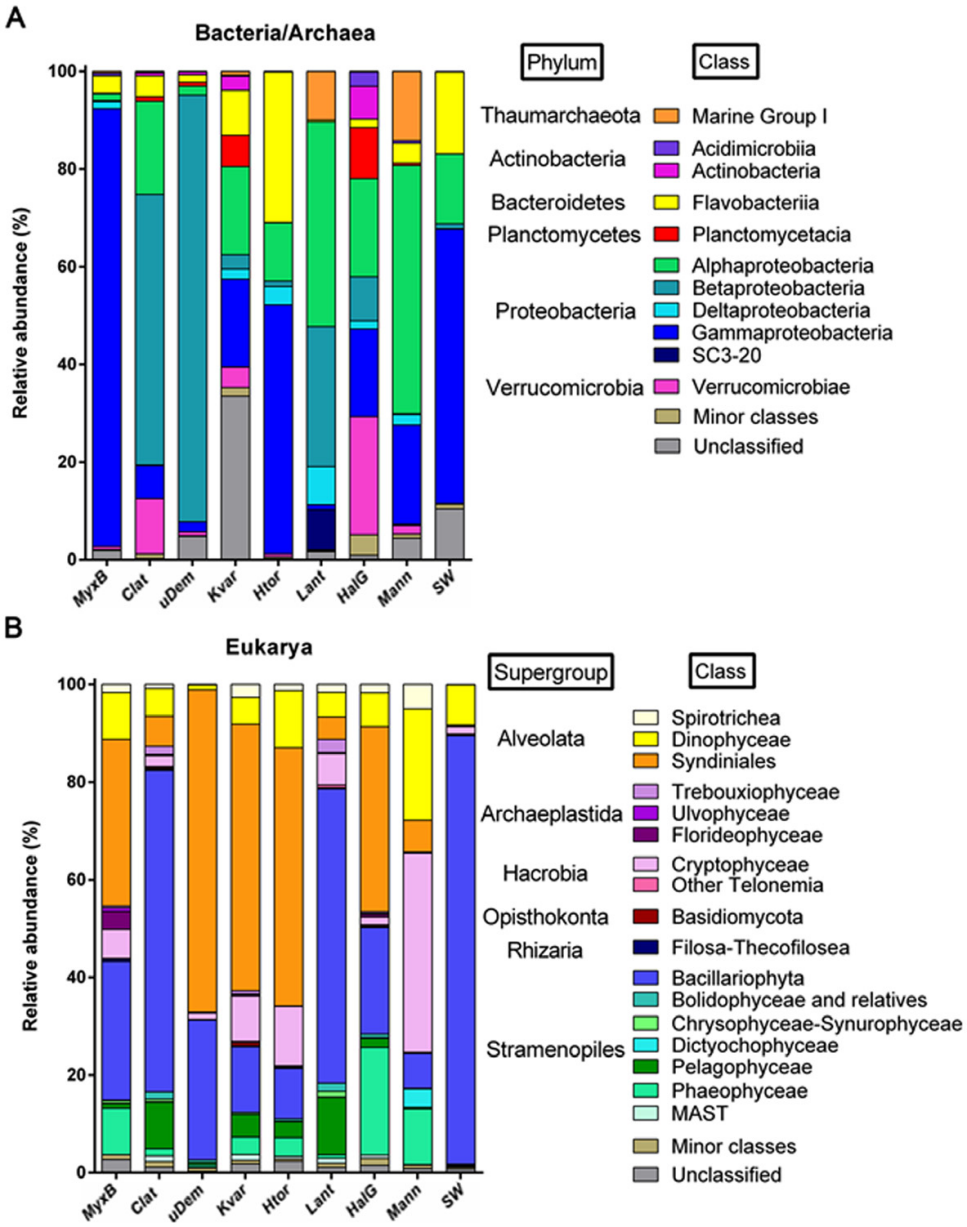
Taxonomic composition. Taxonomic distribution of assigned tag sequences of Antarctic sponge-associated and surrounding seawater (SW) microbial communities. Bars represent relative abundance of sequences belonging to given classes superior to 0.5%. (A) Bacteria/Archaea assigned with the Silva database. (B) Eukaryotes assigned with the PR2 database. *MyxB*: *Myxilla (Burtonanchora)* sp.; *Clat*: *Clathria* sp.; uDem: undetermined Demospongiae; *Kvar*: *Kirkpatrickia variolosa*; *Htor*: *Hymeniacidon torquata*; *Lant*: *Leucetta Antarctica*; *HalG*: *Haliclona (Gellius)* sp.; *Mann*: *Megaciella annectens*; SW: surrounding seawater sample.

A total of 25 bacterial phyla were detected in sponges, and 14 of them were present also in surrounding seawater. In the case of Archaea, the same two phyla were detected in sponges and in seawater (Figure A in S2 Fig). Dominant phyla were Proteobacteria, followed by Bacteroidetes, Verrucomicrobia, Thaumarchaeota and Planctomycetes. Proteobacteria and Bacteroidetes mostly dominated seawater planktonic community. No evidence for photoautotrophic community members was found among bacterial/archaeal taxa, neither in those associated to sponges, nor in those retrieved from the surrounding seawater. In the case of eukaryotes, sequences were classified into eight different supergroups (Figure B in S2 Fig). Dominant supergroups were Stramenopiles, Alveolata and Hacrobia. The seawater planktonic community was dominated by Stramenopiles and Alveolates. At a higher taxonomic resolution level, 13 bacterial/archaeal and 14 eukaryal classes were identified at a relative abundance higher than 0.5% (Fig 2).

Detailed taxonomic composition shows that in the case of Bacteria, sponges *Clathria* sp.and E7 contained a 55% and 87% respectively of sequences assigned to the Order Nitrosomonadales (class Betaproteobacteria). *L*. *antarctica* also contained a considerable proportion of sequences (28%), assigned to Betaproteobacteria belonging to the Order Methylophilales. Finally, *Myxilla (Burtonanchora)* sp. was dominated by sequences assigned to an uncultured Gammaproteobacteria (89.6%) from a Mediterranean sponge clone library (NCBI accession number AJ581351.1) (Fig 2A). In the case of Archaea, the Marine Group I class corresponds to sequences almost exclusively assigned to *Candidatus* Nitrosopumilus. These sequences were detected at high abundances only in sponges *L*. *antarctica* and *M*. *annectens* (9.9 and 14.2% of relative abundance, respectively). In the case of eukaryotes, the most abundant order in most sponges (*Myxilla (Burtonanchora)* sp., E7, *K*. *variolosa*, *H*. *torquata* and *Haliclona (Gellius)* sp.) was the Syndiniales group I, with a mean and s.d. relative abundance of 50 ± 12.9%, followed by sequences assigned to polar centric Mediophyceae (Bacillariophyta) (Fig 2B). These Bacillariophyta sequences were dominating *Clathria* sp. and *L*. *antarctica* (67% and 61%, respectively), and were further classified as *Porosira glacialis*. *Clathria* sp. and *L*. *antarctica* also contained the highest proportion of Pelagophyceae (9.7% and 12%, respectively). *M*. *annectens* contained 40% of sequences assigned to Cryptophyceae, which were further classified in the Cryptomonadales family. This family was detected with a 12% or less relative abundance in all other sponges investigated.

The surrounding planktonic SW community was dominated by sequences assigned to Gammaproteobacteria (56% of relative abundance) followed by Flavobacteria (16.7%), in the case of Bacteria, and by Bacillariophyta (88.5%) and Dinophyceae (8.3%) in the case of Eukarya. Archaeal sequences were almost undetected, with relative abundance lower than 0.05% (Fig 2). S3 and S4 Tables list the indicated relative abundances.

### Similarity pattern within and between sponge-associated and planktonic Antarctic microbial communities

**Fig 3:**
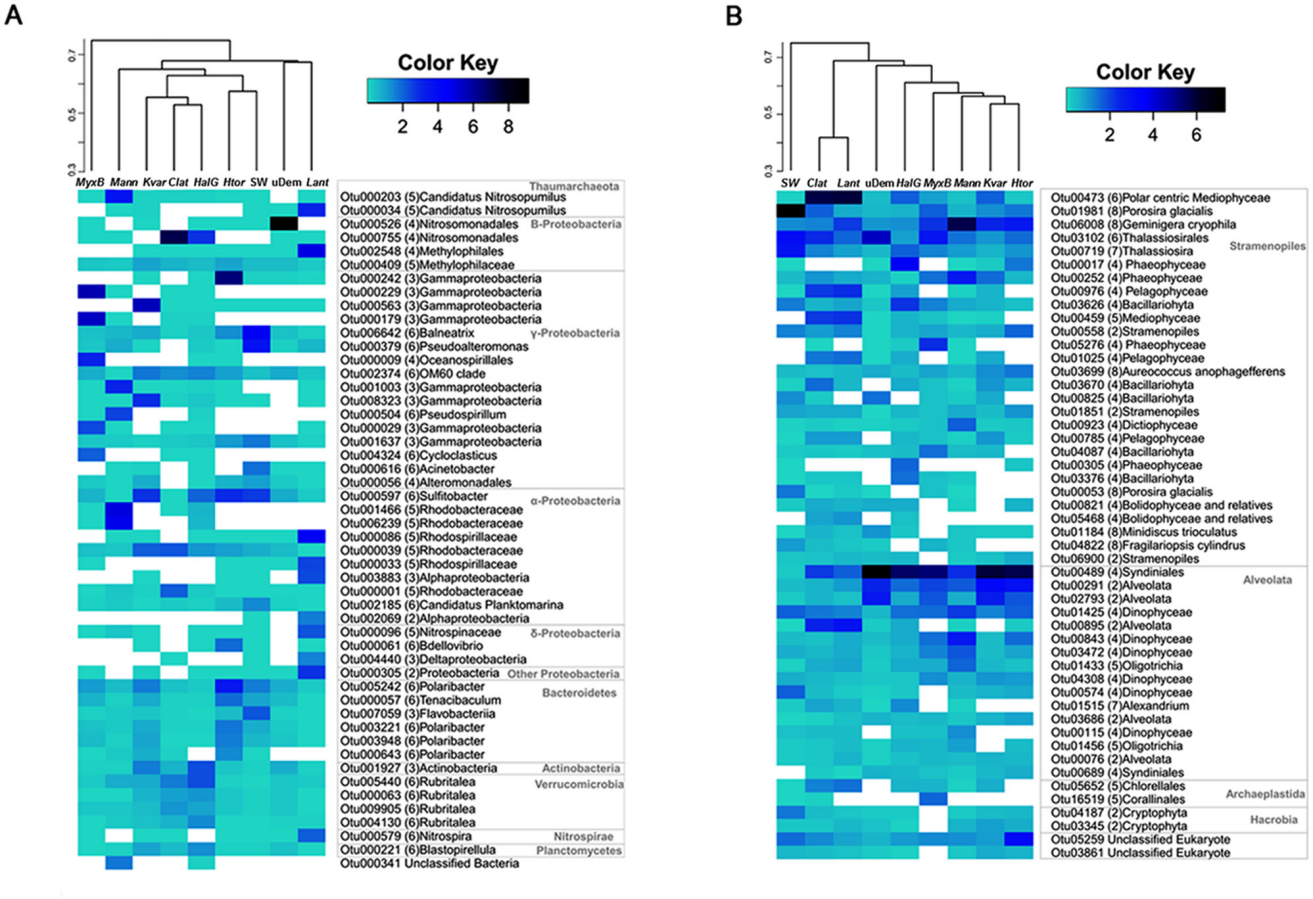
OTU distribution and clustering. Heatmaps representing the relative abundance (50 most abundant OTUs) of bacterial/archaeal (A) and eukaryote (B) taxa associated to Antarctic sponges and the corresponding surrounding seawater microbial communities. Their most resolved taxonomic assignation is included a side each OTU. Numbers represent taxonomic resolution level of the assignation, with (2) = Phylum, (3) = Class, (4) = Order, (5) = Family and (6) = Genus. Cluster above heatmap was generated using weighted Unifrac distance and group average clustering method. Color keys represent square root of relative abundance (in percentage). *MyxB*: *Myxilla (Burtonanchora)* sp.; *Clat*: *Clathria* sp.; uDem: undetermined Demospongiae; *Kvar*: *Kirkpatrickia variolosa*; *Htor*: *Hymeniacidon torquata*; *Lant*: *Leucetta Antarctica*; *HalG*: *Haliclona (Gellius)* sp.; *Mann*: *Megaciella annectens*; SW: surrounding seawater sample.

To compare microbial communities associated to Antarctic sponges among them and with the planktonic surrounding community, OTU-based similarity analyses using weighted Unifrac distances were performed. Similarity patterns do not follow the same trends between bacterial/archaeal and eukaryote communities. Group average clustering shows that bacterial/archaeal communities are at least 30% dissimilar from each other (Fig 3A, cluster). The seawater bacterial/archaeal planktonic community clustered with sponge samples and the community from *Myxilla (Burtonanchora)* sp. stood out as the most dissimilar, with a 73% of dissimilarity. In contrast, eukaryote communities were only 16% dissimilar among them, with a maximum dissimilarity of 61% in the case of the surrounding seawater community (Fig 3B, cluster). Overall, eukaryote communities were 10% more similar among themselves, than bacterial/archaeal communities. Microbial eukaryotes associated to *Clathria* sp. and *L*. *antarctica* were the most homogeneous, albeit over 50% distinct from the communities of all other sponges.

Relative abundances of the 50 most abundant OTUs (representing 77% and 83% of total sequences for Bacteria/Archaea and Eukarya, respectively) and their most resolved taxonomic assignation, are shown in Fig 3. In the case of Bacteria/Archaea, the two most abundant OTUs (OTU000526 and OTU000755), that represented a mean relative abundance and s.d. of 15.9 ± 28.7% among all samples, corresponded to Betaprotebacteria of the order Nitrosomonadales (Fig 3A, heatmap). In the case of Eukarya, the most abundant OTU (OTU00473), representing 21.3 ± 17.5% among all samples, corresponds to the marine group I of Syndiniales class (Fig 3B, heatmap).

### Shared microbiome and host specificity in Antarctic sponges

The abundance of shared OTUs between seawater and sponge microbial communities was calculated. In the case of Bacteria/Archaea, 10.2% of OTUs (with relative abundance >0.1%) were shared between seawater and sponge associated communities. In the case of eukaryotes, 9.6% of OTUs were shared based on the same criteria. Considering only sponge-specific OTUs, core, variable and species-specific sponge microbial communities were determined. In the case of Bacteria/Archaea, 0.3% of OTUs were considered core microbiome (i.e. present in at least 6 sponges), 17.7% were determined as variable community (i.e. present in 2 to 5 sponges), and 57.8% were considered host-specific OTUs (present in only 1 sponge species). In the case of Eukarya, the values observed were 2.3% for core microbiome, 28.3% for the variable community, and 56.6% for host-specific OTUs. Taken together, these results indicate that microbial communities associated to Antarctic sponges share a reduced fraction of OTUs with the seawater community, and, considering the lack of biological replication, suggest that they could be highly host specific within the three domains.

### Antarctic sponge associated bacterial community comparison with sponges from other environments

**Fig 4:**
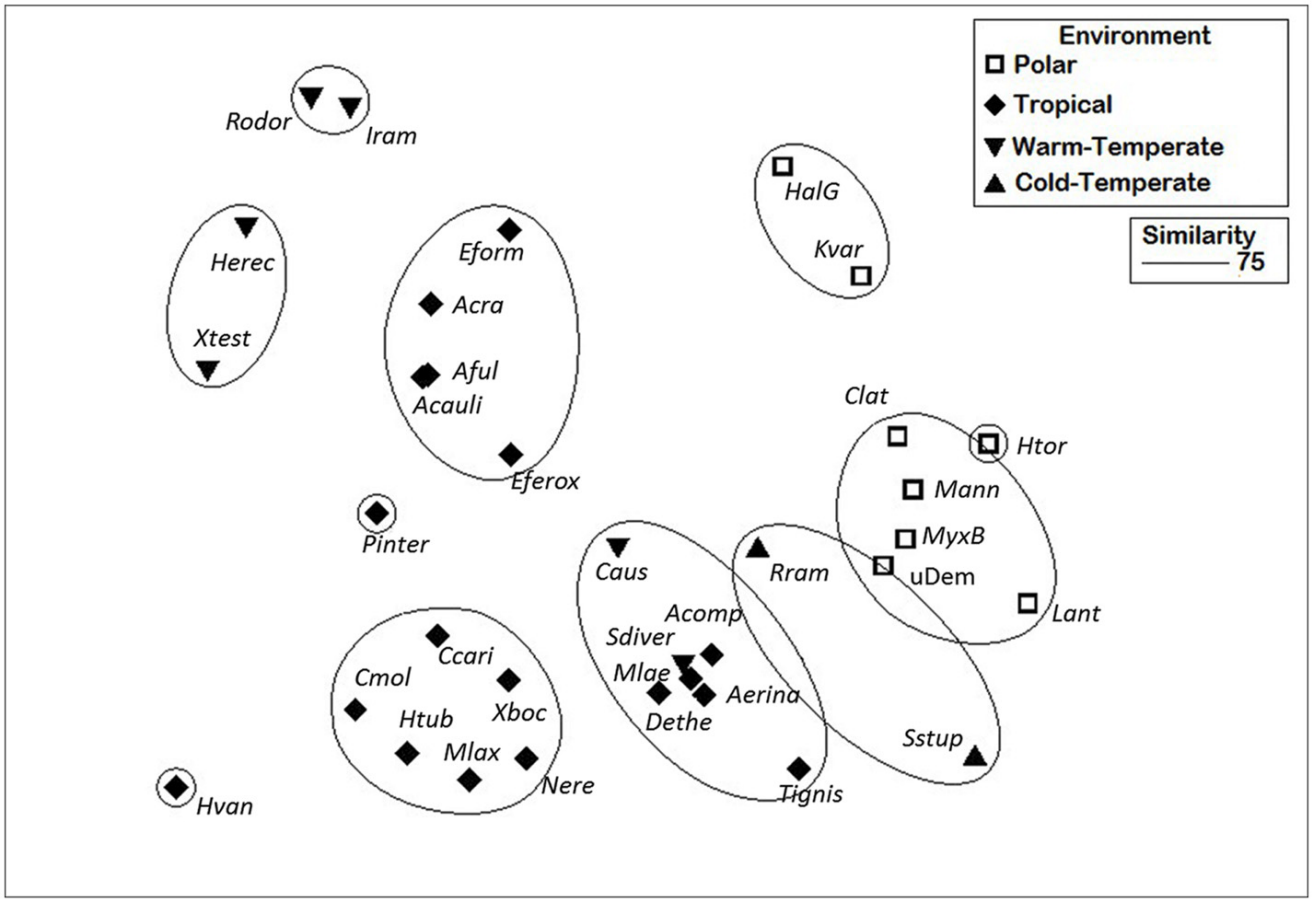
NMDS analysis of bacterial microbiota of sponges from different environments. Analysis is based on Bray-Curtis similarities of relative abundances at phylum-level. Circles indicate similarity level of 75% based on hierarchical cluster analysis. Polar: *MyxB*: *Myxilla (Burtonanchora)* sp.; *Clat*: *Clathria* sp.; uDem: undetermined Demospongiae; *Kvar*: *Kirkpatrickia variolosa*; *Htor*: *Hymeniacidon torquata*; *Lant*: *Leucetta Antarctica*; *HalG*: *Haliclona (Gellius)* sp.; *Mann*: *Megaciella annectens*. Tropical: *Eform*: *Erylus formosus*; *Acra*: *Aiolochroia crassa; Aful*: *Aplysina fulva; Acauli*: *Aplysina cauliformis; Eferox*: *Ectyoplasia ferox; Pinter*: *Placospongia intermedia; Hvan*: *Haliclona vansoesti; Ccari*: *Chondrilla caribensis*; *Cmol*: *Chalinula molitba*; *Htub*: *Haliclona tubifera; Mlax*: *Mycale laxissima*;*Xboc*: *Xestospongia bocatorensis; Nere*: *Niphates erecta*; *Acomp*: *Amphimedon compressa; Mlae*: *Mycale laevis; Aerina*: *Amphimedon erina; Tignis*:*Tedania ignis*; *Dethe*: *Dysidea etheria*. Warm-temperate: *Rodor*: *Rhopaloeides odorabile*; *Iram*: *Ircinia ramosa*; *Herec*: *Hyrtios erectus*; *Xtest*: *Xestospongia testudinaria; Caus*: *Cinachyrella australiensis*, *Sdiver*: *Suberites diversicolor*. Cold-temperate: *Rram*: *Raspailia ramosa*; *Sstup*: *Stelligera stuposa*.

To determine if the bacterial community composition of microorganisms associated to Antarctic sponges was different from those observed elsewhere, phylum level comparisons were made between bacterial 16S rRNA gene inventories from polar (this work), warm-temperate, cold-temperate, and tropical environments [6,9,40,41,42]. NMDS analysis based on Bray-Curtis similarities (Fig 4) has shown a high distinctiveness among different environments (ANOSIM R 0.54, P > 0.001). Bacterial microbiota from Antarctic sponges are highly similar, with cold-temperate environments as the closest relatives, and warm-temperate samples as the most dissimilar. Bacteroidetes, Verrucomicrobiae and Planctomycetes phyla were the most relevant to define Antarctic sponge asscoiated bacterial similarity (SIMPER analysis) with 12%, 8% and 5% of contribution to total Antarctic bacterial community similarity (74%). In addition, using Bray-Curtis distances, Antarctic sponge-associated bacterial communities showed two different general patterns at 75% similarity, with *K*. *variolosa* and *Haliclona (Gellius)* sp. as the most dissimilar. That difference was strongly associated with a high abundance of Planctomycetes.

## Discussion

This work describes the microbial diversity and community composition at three domains level of eight different Antarctic sponges, revealing that bacterial, archaeal and eukaryote symbionts display a high diversity, are different from the seawater microbes and show a particular signature for this ecosystem. Despite the relevance that these holobionts have in the Antarctic benthos, their study has not been extensive, principally due to the difficult access this zone exhibits and that the study of microbial symbiotic relationships is one of the more complicated to achieve. Fortunately, the rapid development in massive sequencing methodologies are allowing the increase of knowledge about diversity, community composition and functionality of sponge-associated microorganism, especially in temperate and tropical areas [6,7,12,42]. In Antarctica, several studies have made descriptions of microbial symbionts of marine sponges, using approximations like microscopy [44], DGGE, clone libraries [20] and cultures [26,27,45]. This is the first study that assesses the microbial communities associated to Antarctic sponges using high throughput sequencing methods.

### Sampling considerations

Considering that Antarctic benthic meiofauna is regarded as vulnerable to future environmental change [46], and the protected status of all the organisms in this continent, we decided to keep sample collection at a minimum. For this reason, this study presents only one individual per species for analyzing microbial communities. Regrettably, the present study considered only one sampling, and elements like temporal or seasonal variation, as well as biological replicates would be needed to confirm if the result reported here reflects a permanent pattern. On one side, the use of several species of Antarctic sponges offers clues about the generality of the results presented here. On the other, the marked proximity of both collecting sites in Fildes Bay (S1 Table), and their consequently similar hydrography is at odds with the goal of illustrating this potential generality. As expected, no differentiation in their microbial sponge-associated communities attributable to sampling site was evident in any of the analyses performed.

### Diversity and taxonomic affiliation of the three-domain symbionts associated to Antarctic sponges

In this study, most rarefaction curves reflect a high coverage of the diversity present in Antarctic sponge microbiomes (S1 Fig). Three of the rarefaction curves (*Myxilla (Burtonanchora) sp*., *Clathria* sp. and *H*. *torquata* in eukaryote dataset) did not reach saturation, probably due to high quantity of Metazoa sequences removed. This is expected due to the intrinsic difficulties to separate microbial eukaryote communities from their host tissues. The number of 97% OTUs obtained for Bacteria/Archaea communities is inside ranges described for Illumina based sequencing of these types of communities [42]. The number of OTUs obtained for eukaryote communities, even after filtration of Metazoa reads, surpass by one order of magnitude a previous effort to characterize fungi and protists associated with marine sponges [21]. Unfortunately, we cannot compare these results with the previous work done in Antarctica, as completely different molecular techniques were used [20].

Most of the studies about sponge-associated microbial communities have considered only one microbial component, and holistic approaches including Bacteria, Archaea and Eukarya at the same time are scarce. The present work provides the most comprehensive characterization of the three-domain microbial diversity associated to (Antarctic) sponges using high throughput sequencing technologies of ribosomal genes. To our knowledge, only one previous study has made approximations to a complete description of the sponge microbial community using this approach [47]. However, they were looking for primers to allow the simultaneous amplification of bacteria and eukaryotes and they analyzed only one sponge from a cold seep site at the Red Sea. Similar approximations, in this case in the Indian Ocean and using a metagenomic (shotgun) approach, described the composition and metabolic profiles of the whole microbial community associated to a deep-sea sponge, indicating that bacterial, archaeal and eukaryotic symbionts have different ecological roles and relationships with sponge host [48].

The present study demonstrated that microorganisms from the three domains of life associated with Antarctic marine sponges display higher diversity than their counterparts from the surrounding SW (Fig 1). Ecological indices show that Bacteria/Archaea associated to three of the sponges display a high diversity (H’>3), which is even greater than the diversity shown by the communities of the surrounding SW (Fig 1 and S2 Table). Altogether, ten more phyla were detected in sponge microbiomes than in seawater (S2 Fig), and this might be considered as an indicator that sponges may be diversity reservoirs in the Antarctic marine ecosystem. In the case of eukaryote communities, the Shannon index was >3 and higher than in SW in seven of the eight sponge microbiomes analyzed. Interestingly, diversity and composition varied among sponge samples following different patterns between the three domains (Figs 2 and 3). This reflects intrinsic differences in how microorganisms from different domains establish symbiotic relationships with these marine animals.

As in most studies about sponge-associated bacteria, Proteobacteria and Bacteroidetes dominated Antarctic sponges. The phylum Proteobacteria has been repeatedly reported as the dominant taxa in association with marine sponges from several locations, with Acidobacteria, Actinobacteria, Chloroflexi, Cyanobacteria and Poribacteria also described as important members [3,5,6,7,40,49,50]. The dominant bacterial assemblages described here are in agreement with the previous report from Antarctic sponges [20]. Our results complement the known bacterial composition through the incorporation of taxa found in minor proportions, increasing to 25 the number of phyla detected in Antarctic sponges. Moreover, our results indicate that the composition of associated bacterial assemblages from Antarctic sponges present differences with sponge symbionts reported on temperate and tropical ecosystems (Figs 2, 3 and S2 Fig), suggesting a particular signature for Antarctic sponges. For instance, the relative abundance of Chloroflexi detected in the Antarctic sponges, is much lower than previously reported in sponges from other environments [6,49,50]. Cyanobacteria, typically described as part of marine sponges, were not detected in Antarctic sponges, which is not surprising considering that Cyanobacteria are typically absent in Antarctic marine waters [51].

Another widely described candidate phylum is Poribacteria, a specific sponge-cluster that has been widely described in marine sponges [14,15] but was not detected in sponges of the present study, nor in studies used here for comparison between environments. Poribacteria have been frequently found in HMA sponges [14,52]. Unfortunately, we are unable to analyze if our Antarctic sponges correspond to HMA or LMA, as no bacterial abundances were estimated. One explanation for the absence of phylum Poribacteria in Antarctic sponges could be related to the extreme environmental conditions, especially the low temperature, as this group of bacteria has only been detected in temperate or tropical sponges. Interestingly, the high abundance of Nitrosomonadales order in two of the sponges, *Clathria* sp. and E7 (Fig 2), is noticeable (Fig 3A). Several bacterial taxa found in the present study are known to be potentially involved in marine biogeochemical cycles, as the case of ammonia-oxidizers Nitrosomonadales and nitrifiers Methylophilales. Our results suggest that bacteria associated to Antarctic sponges could play significant roles in nitrogen conversion in this environment. Further studies are needed to improve the understanding of the functional roles of these microorganisms living as Antarctic sponge symbionts and their contribution to this highly diverse symbiosis.

Archaea have been described to play a dominant role within sponges from different areas like the Mediterranean Sea, cold water and deep-sea environments. These archaeal communities were dominated by Thaumarchaeota, and especially by Cenarcheaceae and Nitrosopumilales families [13,18,53]. In this study, two of the eight sponges (*L*. *antarctica* and *M*. *annectens)* presented relatively high abundances of sequences affiliated to the Nitrosopumilales (Figs 2 and 3). Webster et al. in 2004, found sequences similar to the sponge symbiont *Cenarchaeum symbiosum* (Cenarcheacea family) in the Antarctic sponges *Mycale acerata*, *Latrunculia apicalis* and *K*. *variolosa*. Both of the above mentioned archaeal families are involved in nitrogen cycling activities within sponges, specifically in anaerobic ammonia oxidation [53,54]. Thus, our findings reinforce the prior suggestion that Antarctic sponges could be making an important contribution to the marine nitrogen cycling. Our results also confirm the known distribution of Thaumarachaota to sponges inhabiting Antarctic ecosystem, supporting that this phylum is the major within the Archaea domain in sponges.

The available information about microbial eukaryotes associated with sponges is reduced. It is important to note that taxonomy in eukaryotes has been radically modified in the recent decades in light of new phylogenetic data, and distinct studies use different classifications, so it is sometimes difficult to make comparisons among studies. He et al. in 2014 [21] described 11 phyla within six supergroups, in which Ascomycota, Alveolata and Clorophyta were the major contributors to microbial eukaryotes communities in Chinese sponges. Rhizaria is also an important member of eukaryote sponge symbionts [47]. In the case of Antarctic sponges, the presence of diatoms [19,20,44] and dinoflagellates [20] was previously described. In agreement with these reports, our diatom-assigned sequences are similar to known Antarctic taxa, such as *Thalassiosira antarctica* and *Porosira glacialis* (Fig 3). Furthermore, this work shows the presence of a highly complex microbial eukaryote community within Antarctic sponges. Our results indicate that major contributors to Antarctic sponge-associated microbial eukaryote are Bacillariophyta, Syndiniales and Dinophyceae, with important differences in their composition between distinct sponges (Figs 2 and 3). In general, the eukaryote community was highly dominated by key photosynthetic taxa, like diatoms, but also harbored other phytoplanktonic groups rarely reported, like Pelagophyceae and Phaeophyceae (Figs 2 and 3). The presence of diatoms has been previously described in Antarctic sponges as epibionts, food [19] and parasites [44]. Bacillariophyta also dominated surrounding seawater, however the single Bacillaryophyta OTU dominating the planktonic community is different from the most abundant one in sponges, suggesting that sponge-associated diatoms might also be sponge specific, and possibly playing a key role in carbon metabolism within their hosts, such as Cyanobacteria do in other environments.

Sponges *Haliclona (Gellius)* sp. and *L*. *antarctica* claim our particular attention in terms of their dominance of Phaeophyceae and Pelagophyceae members, respectively. Phaeophyta (brown algae) do not include microbial representatives but marine sponges have shown to live in association with multicellular algae [54], so it is possible that DNA from macroalgal fragments have been extracted together with microbial communities. Another explanation is that, as Phaeophyta display microbial stages in its life cycle [55], the presence of Phaeophyceae sequences in marine sponges could be related to the presence of larvae instead. If this association corresponds to a reservoir before recruitment, or to an incidental association due to sponge pumping and filtration, it is difficult to speculate and further examination would be needed to establish what kind of relationship occurs between sponges and Phaeophyta. Pelagophyceae have been shown to live within Mediterranean sponges [56], but their proportions have not been described. Another photosynthetic taxa described in relatively high abundances in our Antarctic sponges is Cryptophyta. This group is commonly found in Antarctica, and they reached even 40% of relative abundance in one of the sponges of the present study (Fig 2). These photosynthetic members could be also playing a role in carbon metabolism and their virtual absence in seawater could be seen as a preliminary indication that Antarctic sponges could be acting as a diversity reservoir during the summer diatom bloom.

Heterotrophic eukaryotic members affiliated to the Syndiniales group were also present in Antarctic sponges. He and collaborators [21] detected sequences affiliated to Syndiniales, but at very low abundances. Our study shows Syndiniales to be important members of sponge microbiomes for the first time. This group is composed exclusively of marine parasites and has been suggested to infect a variety of marine hosts [57], so they could be acting as parasites inside Antarctic sponge tissues.

Considering that dominant Bacteria, Archaea and Eukarya described here as Antarctic sponge symbionts were previously reported in Webster et al. (2004) and that there is one common sponge species between both studies (*K*. *variolosa*), a temporal stability of these communities could be suggested, at least during Austral summer.

### Shared microbiome and host-specificity in Antarctic sponges and comparison with other environments

An approximation to shared community and host-specificity was performed in the present work. We are aware that our lack of replicates of sponge individuals limits the interpretation of the data presented regarding host-specificity and core microbiome. However, some conclusions can be drawn regarding comparison between Antarctic sponges and seawater microbial communities. Nearly 10% of microbial communities associated with sponges were shared with the surrounding seawater, with similar values between Bacteria/Archaea and Eukarya. In this way, the differences in bacterial composition between sponges and seawater could be a general observation among a variety of habitats, now including the Antarctic ecosystem. Additionally, this could be expanded to the Eukarya domain. Considering the community of Bacteria/Archaea and Eukarya found only in sponges, a little fraction (0.3% and 2.3% respectively) could be classified as “core community”. Schmidt et al. described a reduced percentage of bacterial OTUs common to several sponges [7]. Easson and Thacker corroborated this result in 2014 [42], finding only 1.5% of core bacterial community.

Finally, phylum level comparisons of sponge microbial communities from Antarctica and other areas showed a high degree of difference between environments, with Antarctic sponges clustering together and closely with cold-sea sponges (Fig 4). These analyses were possible only with bacterial communities as no similar data for eukaryotes is available. Our results confirm the differences in composition described before, and shows that although there is a high influence of the host in the differentiation of sponge-associated microbial communities, there could be a certain degree of influence of environmental factors in microbial signatures among different habitats. In this sense, it could be proposed that Antarctic sponges also display a specific microbiome, as showed for deep-sea sponges by Kennedy and collaborators in 2014 [13].

## Conclusion

Results presented here indicate that Antarctic sponges harbor highly diverse microbial communities belonging to three domains of life. Most of the abundant bacteria, archaea and eukaryotes living in symbiosis with Antarctic sponges have been described as significant participants in nitrogen and carbon biogeochemical cycles. In this way, we can suggest that symbioses between microorganisms and Antarctic sponges not only contributes to the nutrition of both parts, but also to Antarctic marine ecosystem. Our work indicates that Antarctic marine sponges harbor a greater microbial diversity than the seawater around them, across the three domains. We found different community composition of symbionts compared to the surrounding planktonic communities, expanding previous observations for Bacteria and including the Antarctic environment. Finally, this work shows that even when sharing an important part of the microbial community with those from other ecosystems, Antarctic sponges have a particular signature in terms of their three-domain diversity and composition. Further functional analysis (e.g. metatranscriptomics), will provide the knowledge needed to fully understand the role that these specific microbiomes have as part of this important marine symbiosis in Antarctica.

## Supporting Information

S1 Fig Chao1 based rarefaction curves of microbial communities associated with different Antarctic sponges and planktonic communities from surrounding seawater (SW). Rarefaction curves were constructed using operational taxonomic units (OTUs) at a 97% sequence similarity.
(TIF)

S2 Fig High level taxonomic distribution of sponge-associated microbial communities. A: Bacterial phyla distribution (only phyla with relative abundance >0.05% are shown). B: Eukaryal supergroups distribution.
(TIF)

S1 Table Environmental parameters of the sampling sites used in this study. (DOCX)

S2 Table Ecological indexes of Antarctic sponge-associated microbial communities. (DOCX)

S3 Table Relative abundance (in percentage) of Phylum, Class and Order of Bacteria and Archaea domains. (DOCX)

S4 Table Relative abundance (in percentage) of Supergroup, Division (Phylum), Class and Order of Eukarya domain. (DOCX)
