# Supplementary figures and images for: Characterization of Bacterial, Archaeal and Eukaryote Symbionts from Antarctic Sponges Reveals a High Diversity at a Three-Domain Level and a Particular Signature for This Ecosystem
Source: PLoS One. 2015 Sep 30;10(9):e0138837. doi: 10.1371/journal.pone.0138837 (PMC4589366; doi:10.1371/journal.pone.0138837)

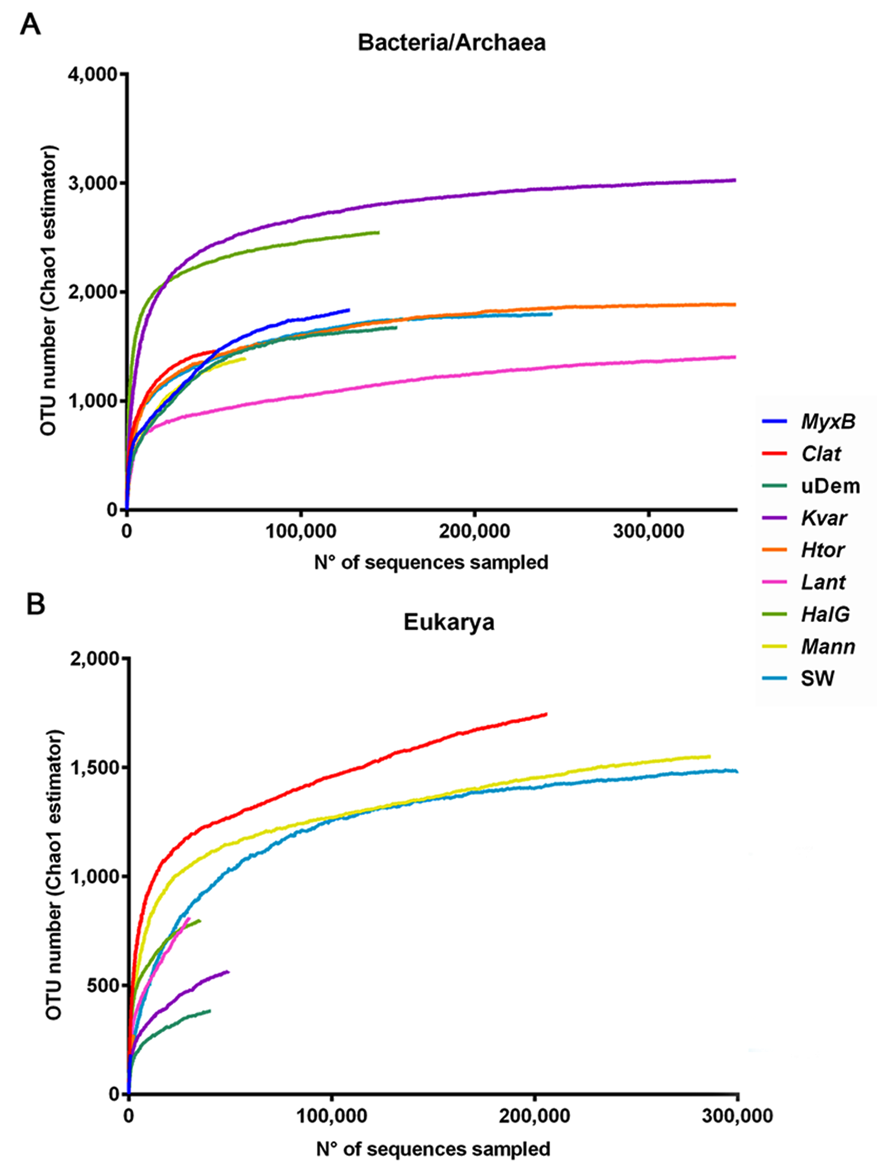

Supplement: S1 Fig — Rarefaction curves were constructed using operational taxonomic units (OTUs) at a 97% sequence similarity. (TIF) [file pone.0138837.s001.tif]

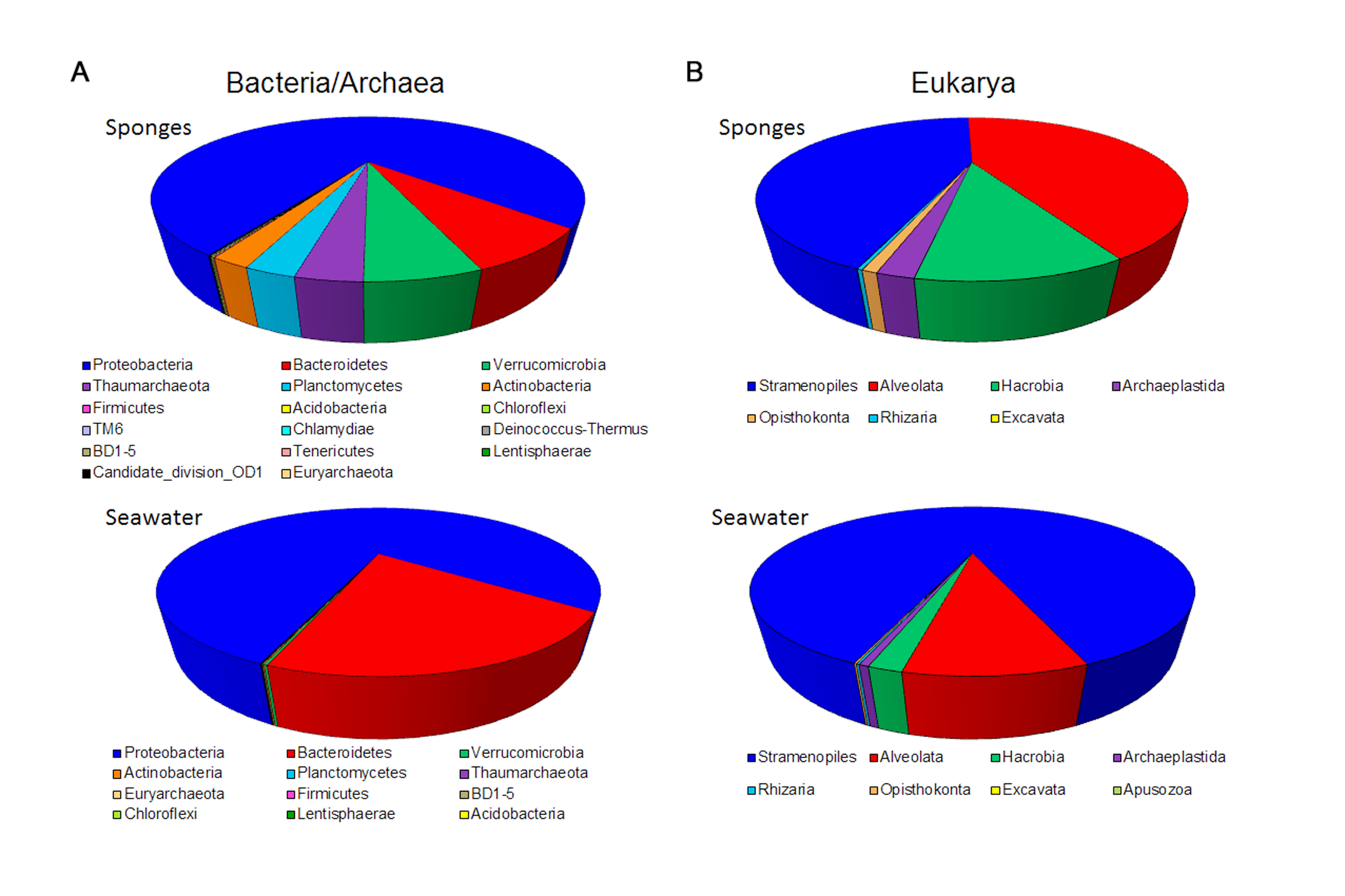

Supplement: S2 Fig — A: Bacterial phyla distribution (only phyla with relative abundance >0.05% are shown). B: Eukaryal supergroups distribution. (TIF) [file pone.0138837.s002.tif]
