# Supplementary material for: Characterization of Bacterial, Archaeal and Eukaryote Symbionts from Antarctic Sponges Reveals a High Diversity at a Three-Domain Level and a Particular Signature for This Ecosystem
Source: PLoS One. 2015 Sep 30;10(9):e0138837. doi: 10.1371/journal.pone.0138837 (PMC4589366; doi:10.1371/journal.pone.0138837)
Supplement: S1 Table — (DOCX) [file pone.0138837.s003.docx]

**S1 Table. Environmental parameters of the sampling sites used in this study.**

| **Site** | **Depth (m)** | **Temp (°C)** | **Salinity (PSU)** | **Fluorescence (mg m^-3^)** | **Dissolved Oxygen (mL L^-1^)** |
| --- | --- | --- | --- | --- | --- |
| **S1** | 5 | 0.84 | 34.1 | 0.392 | 7.917 |
|  | 17 | 0.77 | 34.2 | 1.384 | 7.929 |
|  | 20 | 0.77 | 34.1 | 1.496 | 7.929 |
| **S2** | 5 | 1.0 | 34.2 | 0.624 | 7.884 |
|  | 27 | 0.74 | 34.1 | 1.468 | 7.937 |
