# Supplementary material for: Characterization of Bacterial, Archaeal and Eukaryote Symbionts from Antarctic Sponges Reveals a High Diversity at a Three-Domain Level and a Particular Signature for This Ecosystem
Source: PLoS One. 2015 Sep 30;10(9):e0138837. doi: 10.1371/journal.pone.0138837 (PMC4589366; doi:10.1371/journal.pone.0138837)
Supplement: S2 Table — (DOCX) [file pone.0138837.s004.docx]

**S2 Table. Ecological indexes of Antarctic sponge-associated microbial communities.**

|  | **Bacteria/Archaea** | | | | **Eukarya** | | | |
| --- | --- | --- | --- | --- | --- | --- | --- | --- |
| **Sample** | **Sobs** | **Chao1** | **np-Shannon (H')** | **Simpson (D)** | **Sobs** | **Chao1** | **np-Shannon (H')** | **Simpson (D)** |
| **E4** | 935 | 1,462 | 2.52 | 0.21 | 110 | 183 | 3.45 | 0.10 |
| **E6** | 1,192 | 1,461 | 3.11 | 0.28 | 151 | 368 | 3.54 | 0.13 |
| **E7** | 849 | 1,370 | 1.31 | 0.70 | 59 | 117 | 1.95 | 0.32 |
| **E8** | 1,883 | 2,460 | 3.98 | 0.11 | 100 | 151 | 3.11 | 0.15 |
| **E9** | 1,016 | 1,418 | 2.70 | 0.22 | 104 | 197 | 3.10 | 0.14 |
| **E10** | 716 | 926 | 2.61 | 0.13 | 133 | 246 | 3.36 | 0.14 |
| **E11** | 2,063 | 2,301 | 5.61 | 0.02 | 172 | 324 | 3.73 | 0.12 |
| **E12** | 904 | 1,320 | 2.90 | 0.15 | 117 | 281 | 3.26 | 0.13 |
| **SW** | 1,092 | 1,401 | 3.80 | 0.08 | 69 | 151 | 2.24 | 0.31 |
