# Supplementary material for: Characterization of Bacterial, Archaeal and Eukaryote Symbionts from Antarctic Sponges Reveals a High Diversity at a Three-Domain Level and a Particular Signature for This Ecosystem
Source: PLoS One. 2015 Sep 30;10(9):e0138837. doi: 10.1371/journal.pone.0138837 (PMC4589366; doi:10.1371/journal.pone.0138837)
Supplement: S3 Table — (DOCX) [file pone.0138837.s005.docx]

**S3 Table. Relative abundance (in percentage) of Phylum, Class and Order of Bacteria and Archaea domains.**

|  | ***Myxilla (Burtonanchora)* sp.** | ***Clathria* sp.** | **Un. Demospongiae** | ***Kirkpatrickia variolosa*** | ***Hymeniacidon torquata*** | ***Leucetta antarctica*** | ***Haliclona (Gellius)* sp.** | ***Megaciella annectens*** | **SW** |
| --- | --- | --- | --- | --- | --- | --- | --- | --- | --- |
| **Phylum** |  |  |  |  |  |  |  |  |  |
| Euryarchaeota | 0.00 | 0.00 | 0.00 | 0.001 | 0.00 | 0.00 | 0.00 | 0.00 | 0.02 |
| Thaumarchaeota | 0.34 | 0.03 | 0.01 | 0.78 | 0.01 | 9.91 | 0.11 | 14.20 | 0.02 |
| Acidobacteria | 0.01 | 0.01 | 0.00 | 0.37 | 0.00 | 0.01 | 0.19 | 0.04 | 0.00 |
| Actinobacteria | 0.54 | 0.86 | 0.69 | 3.12 | 0.11 | 0.03 | 9.91 | 0.51 | 0.07 |
| BD1-5 | 0.001 | 0.00 | 0.00 | 0.00 | 0.001 | 0.00 | 0.20 | 0.001 | 0.00 |
| Bacteroidetes | 3.64 | 4.43 | 1.58 | 9.71 | 31.01 | 0.44 | 2.10 | 4.49 | 17.41 |
| Candidate division BRC1 | 0.00 | 0.00 | 0.00 | 0.005 | 0.00 | 0.00 | 0.00 | 0.00 | 0.00 |
| Candidate division OD1 | 0.00 | 0.00 | 0.00 | 0.001 | 0.00 | 0.00 | 0.05 | 0.003 | 0.00 |
| Chlamydiae | 0.005 | 0.03 | 0.001 | 0.02 | 0.01 | 0.00 | 0.26 | 0.02 | 0.00 |
| Chlorobi | 0.01 | 0.00 | 0.00 | 0.02 | 0.00 | 0.00 | 0.00 | 0.004 | 0.00 |
| Chloroflexi | 0.01 | 0.01 | 0.001 | 0.06 | 0.001 | 0.001 | 0.53 | 0.01 | 0.001 |
| Cyanobacteria | 0.01 | 0.25 | 0.01 | 0.70 | 0.03 | 0.01 | 0.12 | 0.00 | 2.92 |
| Deferribacteres | 0.00 | 0.00 | 0.00 | 0.00 | 0.003 | 0.00 | 0.00 | 0.00 | 0.02 |
| Deinococcus-Thermus | 0.004 | 0.00 | 0.00 | 0.02 | 0.00 | 0.00 | 0.18 | 0.00 | 0.00 |
| Fibrobacteres | 0.00 | 0.00 | 0.00 | 0.002 | 0.001 | 0.00 | 0.00 | 0.00 | 0.001 |
| Firmicutes | 0.00 | 0.11 | 0.04 | 0.13 | 0.01 | 0.01 | 0.33 | 0.01 | 0.02 |
| Fusobacteria | 0.002 | 0.01 | 0.003 | 0.01 | 0.02 | 0.01 | 0.02 | 0.00 | 0.05 |
| Gemmatimonadetes | 0.001 | 0.00 | 0.00 | 0.05 | 0.00 | 0.00 | 0.00 | 0.00 | 0.00 |
| Lentisphaerae | 0.003 | 0.004 | 0.00 | 0.02 | 0.004 | 0.00 | 0.06 | 0.00 | 0.001 |
| Nitrospirae | 0.00 | 0.00 | 0.00 | 0.002 | 0.001 | 0.00 | 0.001 | 0.004 | 0.00 |
| Planctomycetes | 0.14 | 1.01 | 0.80 | 6.58 | 0.10 | 0.03 | 10.67 | 0.50 | 0.04 |
| Proteobacteria | 94.56 | 81.53 | 96.10 | 73.30 | 67.96 | 87.68 | 49.23 | 75.04 | 71.78 |
| SHA-109 | 0.00 | 0.00 | 0.001 | 0.01 | 0.00 | 0.00 | 0.02 | 0.004 | 0.00 |
| Spirochaetae | 0.00 | 0.00 | 0.00 | 0.00 | 0.00 | 0.00 | 0.01 | 0.00 | 0.001 |
| TM6 | 0.02 | 0.002 | 0.01 | 0.08 | 0.002 | 0.001 | 0.41 | 0.03 | 0.00 |
| Tenericutes | 0.00 | 0.02 | 0.00 | 0.002 | 0.00 | 0.00 | 0.12 | 0.001 | 0.00 |
| Verrucomicrobia | 0.63 | 11.46 | 0.73 | 4.32 | 0.59 | 0.31 | 24.79 | 1.98 | 0.15 |
| WCHB1-60 | 0.00 | 0.00 | 0.001 | 0.03 | 0.00 | 0.00 | 0.001 | 0.00 | 0.00 |
| unclassified Bacteria/Archaea | 0.06 | 0.26 | 0.02 | 0.68 | 0.14 | 1.59 | 0.68 | 3.14 | 7.49 |
| **Class** |  |  |  |  |  |  |  |  |  |
| Thermoplasmata | 0.000 | 0.000 | 0.000 | 0.001 | 0.000 | 0.000 | 0.000 | 0.000 | 0.020 |
| Marine Group I (Thaumarchaeota) | 0.337 | 0.026 | 0.011 | 0.777 | 0.011 | 9.905 | 0.105 | 14.197 | 0.022 |
| Acidobacteria | 0.007 | 0.008 | 0.003 | 0.250 | 0.001 | 0.006 | 0.096 | 0.035 | 0.000 |
| Holophagae | 0.003 | 0.000 | 0.000 | 0.122 | 0.000 | 0.000 | 0.090 | 0.004 | 0.000 |
| Acidimicrobiia | 0.489 | 0.239 | 0.051 | 0.189 | 0.014 | 0.000 | 2.949 | 0.480 | 0.017 |
| Actinobacteria | 0.049 | 0.611 | 0.634 | 2.919 | 0.097 | 0.024 | 6.700 | 0.026 | 0.055 |
| unclass. Actinobacteria | 0.000 | 0.006 | 0.001 | 0.010 | 0.003 | 0.000 | 0.176 | 0.007 | 0.000 |
| unclass. BD1-5 | 0.001 | 0.000 | 0.000 | 0.000 | 0.001 | 0.000 | 0.200 | 0.001 | 0.004 |
| Cytophagia | 0.013 | 0.092 | 0.007 | 0.253 | 0.051 | 0.040 | 0.060 | 0.022 | 0.284 |
| Flavobacteriia | 3.567 | 4.301 | 1.547 | 9.164 | 30.818 | 0.373 | 1.791 | 4.109 | 16.750 |
| Sphingobacteriia | 0.034 | 0.023 | 0.009 | 0.167 | 0.040 | 0.014 | 0.039 | 0.338 | 0.136 |
| unclass. Bacteroidetes | 0.023 | 0.009 | 0.004 | 0.064 | 0.086 | 0.008 | 0.210 | 0.025 | 0.222 |
| Chlamydiae | 0.005 | 0.028 | 0.001 | 0.019 | 0.009 | 0.000 | 0.264 | 0.021 | 0.000 |
| Anaerolineae | 0.009 | 0.002 | 0.000 | 0.040 | 0.001 | 0.001 | 0.270 | 0.000 | 0.001 |
| KD4-96 | 0.002 | 0.008 | 0.001 | 0.015 | 0.000 | 0.000 | 0.226 | 0.000 | 0.000 |
| Deinococci | 0.004 | 0.000 | 0.000 | 0.022 | 0.000 | 0.000 | 0.183 | 0.000 | 0.000 |
| Bacilli | 0.000 | 0.074 | 0.000 | 0.001 | 0.000 | 0.003 | 0.139 | 0.001 | 0.009 |
| Clostridia | 0.005 | 0.017 | 0.029 | 0.073 | 0.006 | 0.005 | 0.179 | 0.006 | 0.007 |
| OM190 | 0.001 | 0.011 | 0.004 | 0.024 | 0.002 | 0.000 | 0.165 | 0.012 | 0.002 |
| Planctomycetacia | 0.130 | 0.922 | 0.788 | 6.467 | 0.081 | 0.031 | 10.386 | 0.459 | 0.040 |
| Alphaproteobacteria | 1.400 | 19.081 | 1.815 | 17.973 | 11.888 | 41.931 | 20.120 | 50.821 | 14.312 |
| Betaproteobacteria | 0.203 | 55.411 | 87.373 | 2.974 | 1.151 | 28.689 | 9.021 | 0.144 | 1.050 |
| Deltaproteobacteria | 1.508 | 0.100 | 0.015 | 2.078 | 3.763 | 7.783 | 1.700 | 2.168 | 0.081 |
| Epsilonproteobacteria | 0.012 | 0.138 | 0.016 | 0.062 | 0.067 | 0.002 | 0.226 | 0.003 | 0.175 |
| Gammaproteobacteria | 89.608 | 6.744 | 2.087 | 17.999 | 50.990 | 1.030 | 17.873 | 20.299 | 56.135 |
| SC3-20 | 0.007 | 0.023 | 0.003 | 0.031 | 0.002 | 8.198 | 0.008 | 0.288 | 0.005 |
| unclass. Proteobacteria | 1.822 | 0.030 | 4.791 | 32.153 | 0.096 | 0.040 | 0.261 | 1.282 | 0.017 |
| unclass. TM6 | 0.025 | 0.002 | 0.006 | 0.077 | 0.002 | 0.001 | 0.413 | 0.034 | 0.000 |
| Mollicutes | 0.000 | 0.017 | 0.000 | 0.002 | 0.000 | 0.000 | 0.117 | 0.001 | 0.000 |
| Opitutae | 0.005 | 0.098 | 0.002 | 0.012 | 0.004 | 0.002 | 0.119 | 0.238 | 0.004 |
| Spartobacteria | 0.012 | 0.000 | 0.004 | 0.116 | 0.003 | 0.001 | 0.479 | 0.000 | 0.001 |
| Verrucomicrobiae | 0.610 | 11.360 | 0.726 | 4.181 | 0.578 | 0.203 | 24.188 | 1.746 | 0.144 |
| unclass. Verrucomicrobia | 0.000 | 0.000 | 0.000 | 0.007 | 0.001 | 0.101 | 0.000 | 0.000 | 0.000 |
| **Order** |  |  |  |  |  |  |  |  |  |
| Thermoplasmatales | 0.000 | 0.000 | 0.000 | 0.001 | 0.000 | 0.000 | 0.000 | 0.000 | 0.020 |
| Unclass. Archaea | 0.000 | 0.000 | 0.000 | 0.199 | 0.001 | 0.000 | 0.000 | 0.000 | 0.000 |
| Subgroup_9 | 0.002 | 0.000 | 0.000 | 0.148 | 0.000 | 0.000 | 0.026 | 0.000 | 0.000 |
| Subgroup_10 | 0.003 | 0.000 | 0.000 | 0.122 | 0.000 | 0.000 | 0.090 | 0.004 | 0.000 |
| Acidimicrobiales | 0.489 | 0.239 | 0.051 | 0.189 | 0.014 | 0.000 | 2.949 | 0.480 | 0.017 |
| Corynebacteriales | 0.002 | 0.019 | 0.000 | 0.018 | 0.000 | 0.000 | 0.221 | 0.000 | 0.007 |
| Micrococcales | 0.009 | 0.077 | 0.021 | 0.126 | 0.007 | 0.000 | 0.500 | 0.007 | 0.007 |
| PeM15 | 0.037 | 0.505 | 0.607 | 2.762 | 0.090 | 0.023 | 5.906 | 0.019 | 0.041 |
| Cytophagales | 0.012 | 0.091 | 0.006 | 0.238 | 0.051 | 0.039 | 0.048 | 0.022 | 0.284 |
| Flavobacteriales | 3.567 | 4.301 | 1.547 | 9.164 | 30.818 | 0.373 | 1.791 | 4.109 | 16.750 |
| Sphingobacteriales | 0.034 | 0.023 | 0.009 | 0.167 | 0.040 | 0.014 | 0.039 | 0.338 | 0.136 |
| Chlamydiales | 0.005 | 0.028 | 0.001 | 0.019 | 0.009 | 0.000 | 0.264 | 0.021 | 0.000 |
| Anaerolineales | 0.009 | 0.002 | 0.000 | 0.040 | 0.001 | 0.001 | 0.270 | 0.000 | 0.001 |
| SubsectionI | 0.000 | 0.241 | 0.000 | 0.000 | 0.001 | 0.000 | 0.024 | 0.003 | 0.003 |
| Deinococcales | 0.004 | 0.000 | 0.000 | 0.022 | 0.000 | 0.000 | 0.183 | 0.000 | 0.000 |
| Bacillales | 0.000 | 0.072 | 0.000 | 0.001 | 0.000 | 0.002 | 0.108 | 0.001 | 0.008 |
| Clostridiales | 0.005 | 0.017 | 0.029 | 0.073 | 0.006 | 0.005 | 0.179 | 0.006 | 0.007 |
| Planctomycetales | 0.130 | 0.922 | 0.788 | 6.467 | 0.081 | 0.031 | 10.386 | 0.459 | 0.040 |
| Caulobacterales | 0.036 | 0.002 | 0.008 | 0.033 | 0.041 | 0.043 | 0.012 | 0.069 | 0.163 |
| OCS116_clade | 0.004 | 0.109 | 0.006 | 0.087 | 0.019 | 6.154 | 0.192 | 0.019 | 0.024 |
| Rhizobiales | 0.104 | 0.424 | 0.153 | 0.941 | 0.031 | 0.025 | 3.800 | 0.229 | 0.291 |
| Rhodobacterales | 0.989 | 17.614 | 1.551 | 16.058 | 10.778 | 0.140 | 14.352 | 49.711 | 11.969 |
| Rhodospirillales | 0.037 | 0.060 | 0.016 | 0.175 | 0.101 | 29.480 | 0.219 | 0.175 | 0.532 |
| Rickettsiales | 0.040 | 0.089 | 0.005 | 0.023 | 0.066 | 1.516 | 0.073 | 0.026 | 0.512 |
| SAR11_clade | 0.002 | 0.004 | 0.001 | 0.005 | 0.077 | 0.004 | 0.015 | 0.006 | 0.600 |
| Sphingomonadales | 0.111 | 0.132 | 0.001 | 0.013 | 0.018 | 0.452 | 0.261 | 0.322 | 0.158 |
| Burkholderiales | 0.005 | 0.126 | 0.049 | 1.686 | 0.030 | 0.008 | 0.309 | 0.007 | 0.344 |
| Methylophilales | 0.143 | 0.507 | 0.283 | 1.062 | 1.109 | 24.276 | 0.326 | 0.117 | 0.693 |
| Nitrosomonadales | 0.035 | 54.501 | 84.377 | 0.199 | 0.008 | 0.012 | 8.351 | 0.012 | 0.009 |
| Bdellovibrionales | 0.078 | 0.034 | 0.002 | 0.824 | 3.757 | 0.009 | 0.092 | 1.798 | 0.004 |
| Desulfobacterales | 1.381 | 0.028 | 0.009 | 0.055 | 0.002 | 5.302 | 0.244 | 0.112 | 0.014 |
| SAR324_clade(Marine_group_B) | 0.000 | 0.004 | 0.001 | 0.000 | 0.001 | 2.464 | 0.000 | 0.000 | 0.058 |
| Sh765B-TzT-29 | 0.045 | 0.009 | 0.001 | 1.168 | 0.001 | 0.002 | 1.190 | 0.250 | 0.002 |
| Campylobacterales | 0.012 | 0.138 | 0.016 | 0.062 | 0.067 | 0.002 | 0.226 | 0.003 | 0.175 |
| Alteromonadales | 1.123 | 3.305 | 1.019 | 4.012 | 2.443 | 0.719 | 6.394 | 1.540 | 19.581 |
| Chromatiales | 0.048 | 0.040 | 0.004 | 0.068 | 0.020 | 0.006 | 0.148 | 0.213 | 0.080 |
| E01-9C-26_marine_group | 66.285 | 0.124 | 0.017 | 8.382 | 44.189 | 0.011 | 0.580 | 9.726 | 0.026 |
| EC3 | 0.002 | 0.000 | 0.000 | 0.011 | 0.001 | 0.000 | 0.104 | 0.003 | 0.000 |
| Enterobacteriales | 0.004 | 0.202 | 0.003 | 0.003 | 0.004 | 0.012 | 0.081 | 0.004 | 0.002 |
| Gammaproteobacteria_Incertae_Sedis | 0.073 | 0.083 | 0.003 | 0.015 | 0.007 | 0.011 | 0.078 | 0.113 | 0.023 |
| KI89A_clade | 0.002 | 0.032 | 0.001 | 0.001 | 0.000 | 0.000 | 0.003 | 0.364 | 0.015 |
| Legionellales | 0.005 | 0.145 | 0.037 | 0.424 | 0.007 | 0.004 | 2.829 | 0.010 | 0.002 |
| NKB5 | 0.005 | 0.043 | 0.013 | 0.076 | 0.008 | 0.001 | 0.425 | 0.007 | 0.004 |
| Oceanospirillales | 16.606 | 0.690 | 0.362 | 1.204 | 3.457 | 0.107 | 0.534 | 6.947 | 28.906 |
| Order_Incertae_Sedis | 0.063 | 0.711 | 0.028 | 0.027 | 0.028 | 0.018 | 0.964 | 0.386 | 0.056 |
| Pseudomonadales | 0.018 | 0.272 | 0.029 | 0.451 | 0.006 | 0.047 | 0.242 | 0.138 | 6.717 |
| Sva0071 | 0.000 | 0.017 | 0.000 | 0.002 | 0.000 | 0.000 | 0.133 | 0.000 | 0.000 |
| Thiotrichales | 4.479 | 0.341 | 0.177 | 2.027 | 0.292 | 0.032 | 0.505 | 0.185 | 0.366 |
| Vibrionales | 0.000 | 0.015 | 0.002 | 0.002 | 0.001 | 0.002 | 0.001 | 0.000 | 0.183 |
| Xanthomonadales | 0.027 | 0.038 | 0.006 | 0.099 | 0.015 | 0.008 | 0.347 | 0.323 | 0.064 |
| Mycoplasmatales | 0.000 | 0.017 | 0.000 | 0.002 | 0.000 | 0.000 | 0.117 | 0.001 | 0.000 |
| Chthoniobacterales | 0.012 | 0.000 | 0.004 | 0.116 | 0.003 | 0.001 | 0.479 | 0.000 | 0.001 |
| Verrucomicrobiales | 0.610 | 11.360 | 0.726 | 4.181 | 0.578 | 0.203 | 24.188 | 1.746 | 0.144 |
| Unclassified taxa | 3.235 | 1.929 | 7.963 | 36.365 | 1.606 | 28.387 | 8.222 | 19.757 | 10.812 |

For Class and Order, only taxa above 0.1% are indicated. Classification is according to Silva database v119.
