# Supplementary material for: Characterization of Bacterial, Archaeal and Eukaryote Symbionts from Antarctic Sponges Reveals a High Diversity at a Three-Domain Level and a Particular Signature for This Ecosystem
Source: PLoS One. 2015 Sep 30;10(9):e0138837. doi: 10.1371/journal.pone.0138837 (PMC4589366; doi:10.1371/journal.pone.0138837)
Supplement: S4 Table — (DOCX) [file pone.0138837.s006.docx]

**S4 Table. Relative abundance (in percentage) of Supergroup, Division (Phylum), Class and Order of Eukarya domain.**

|  | ***Myxilla (Burtonanchora)* sp.** | ***Clathria* sp.** | **Un. Demospongiae** | ***Kirkpatrickia variolosa*** | ***Hymeniacidon torquata*** | ***Leucetta antarctica*** | ***Haliclona (Gellius)* sp.** | ***Megaciella annectens*** | **SW** |
| --- | --- | --- | --- | --- | --- | --- | --- | --- | --- |
| **Supergroup** |  |  |  |  |  |  |  |  |  |
| Alveolata | 38.70 | 11.07 | 55.71 | 42.39 | 43.42 | 9.08 | 39.37 | 29.20 | 7.92 |
| Apusozoa | 0.00 | 0.00 | 0.00 | 0.00 | 0.00 | 0.00 | 0.00 | 0.00 | 0.00 |
| Archaeplastida | 4.41 | 2.08 | 0.21 | 0.78 | 0.17 | 2.60 | 1.21 | 0.18 | 0.45 |
| Excavata | 0.00 | 0.01 | 0.00 | 0.00 | 0.00 | 0.00 | 0.04 | 0.13 | 0.01 |
| Hacrobia | 5.07 | 2.09 | 1.05 | 6.58 | 8.01 | 5.54 | 1.53 | 34.40 | 1.52 |
| Opisthokonta | 2.21 | 0.29 | 0.24 | 0.69 | 0.17 | 0.10 | 0.87 | 0.28 | 0.11 |
| Rhizaria | 0.44 | 0.40 | 0.04 | 0.09 | 0.13 | 0.28 | 0.09 | 0.13 | 0.13 |
| Stramenopiles | 33.52 | 67.36 | 24.97 | 16.16 | 13.03 | 61.72 | 39.78 | 19.78 | 82.64 |
| unclassified eukaryote | 15.66 | 16.71 | 17.78 | 33.30 | 35.06 | 20.69 | 17.11 | 15.89 | 7.22 |
| **Division** |  |  |  |  |  |  |  |  |  |
| Ciliophora | 1.764 | 0.818 | 0.429 | 1.828 | 1.342 | 1.335 | 1.759 | 4.466 | 0.095 |
| Dinophyta | 36.935 | 9.995 | 55.167 | 40.149 | 41.948 | 7.733 | 37.202 | 24.704 | 7.805 |
| unclass. Alveolata | 0.000 | 0.250 | 0.127 | 0.414 | 0.130 | 0.017 | 0.423 | 0.049 | 0.016 |
| Chlorophyta | 1.323 | 1.972 | 0.144 | 0.563 | 0.130 | 2.506 | 0.758 | 0.055 | 0.311 |
| Rhodophyta | 3.087 | 0.092 | 0.040 | 0.216 | 0.043 | 0.087 | 0.431 | 0.125 | 0.124 |
| Discoba | 0.000 | 0.008 | 0.000 | 0.000 | 0.000 | 0.000 | 0.037 | 0.127 | 0.007 |
| Cryptophyta | 4.961 | 1.877 | 1.035 | 6.175 | 7.922 | 5.079 | 1.320 | 34.340 | 1.381 |
| Haptophyta | 0.000 | 0.000 | 0.000 | 0.198 | 0.000 | 0.000 | 0.000 | 0.000 | 0.000 |
| Telonemia | 0.110 | 0.215 | 0.015 | 0.210 | 0.087 | 0.434 | 0.216 | 0.078 | 0.141 |
| Fungi | 0.000 | 0.271 | 0.223 | 0.513 | 0.130 | 0.087 | 0.522 | 0.131 | 0.011 |
| unclass. Opisthokonta | 2.205 | 0.017 | 0.020 | 0.182 | 0.043 | 0.010 | 0.346 | 0.150 | 0.096 |
| Cercozoa | 0.441 | 0.392 | 0.045 | 0.093 | 0.130 | 0.279 | 0.079 | 0.078 | 0.131 |
| Ochrophyta | 33.407 | 65.763 | 24.784 | 14.741 | 11.688 | 59.977 | 38.817 | 19.157 | 81.911 |
| unclass. Stramenopiles | 0.110 | 1.619 | 0.191 | 1.419 | 1.342 | 1.759 | 0.979 | 0.637 | 0.750 |
| **Class** |  |  |  |  |  |  |  |  |  |
| Litostomatea | 0.000 | 0.009 | 0.000 | 0.024 | 0.000 | 0.000 | 0.079 | 0.159 | 0.000 |
| Phyllopharyngea | 0.392 | 0.042 | 0.335 | 0.079 | 0.200 | 0.000 | 0.164 | 0.083 | 0.001 |
| Spirotrichea | 1.699 | 0.874 | 0.121 | 2.638 | 1.333 | 1.679 | 1.738 | 5.039 | 0.096 |
| unclass. Ciliophora | 0.000 | 0.043 | 0.051 | 0.000 | 0.533 | 0.004 | 0.062 | 0.014 | 0.002 |
| Dinophyceae | 9.542 | 5.659 | 1.050 | 5.518 | 11.667 | 4.978 | 6.961 | 22.748 | 8.227 |
| Syndiniales | 34.118 | 6.156 | 65.967 | 54.585 | 52.867 | 4.567 | 37.869 | 6.573 | 0.153 |
| unclass. Dinophyta | 0.131 | 0.181 | 0.063 | 0.088 | 0.067 | 0.204 | 0.034 | 0.029 | 0.030 |
| Mamiellophyceae | 0.392 | 0.454 | 0.015 | 0.091 | 0.200 | 0.212 | 0.352 | 0.003 | 0.244 |
| Trebouxiophyceae | 0.392 | 1.708 | 0.000 | 0.656 | 0.000 | 2.777 | 0.373 | 0.016 | 0.002 |
| Ulvophyceae | 0.784 | 0.096 | 0.160 | 0.097 | 0.000 | 0.089 | 0.185 | 0.046 | 0.074 |
| Florideophyceae | 3.660 | 0.110 | 0.048 | 0.324 | 0.067 | 0.110 | 0.520 | 0.148 | 0.134 |
| Euglenozoa | 0.000 | 0.009 | 0.000 | 0.000 | 0.000 | 0.000 | 0.044 | 0.151 | 0.008 |
| Cryptophyceae | 5.882 | 2.253 | 1.258 | 9.257 | 12.200 | 6.403 | 1.591 | 40.800 | 1.488 |
| Prymnesiophyceae | 0.000 | 0.000 | 0.000 | 0.296 | 0.000 | 0.000 | 0.000 | 0.000 | 0.000 |
| unclass. Telonemia | 0.131 | 0.258 | 0.018 | 0.315 | 0.133 | 0.547 | 0.260 | 0.093 | 0.152 |
| Ascomycota | 0.000 | 0.045 | 0.223 | 0.003 | 0.000 | 0.034 | 0.363 | 0.117 | 0.000 |
| Basidiomycota | 0.000 | 0.272 | 0.030 | 0.663 | 0.200 | 0.076 | 0.267 | 0.038 | 0.012 |
| unclass. Fungi | 2.614 | 0.020 | 0.024 | 0.272 | 0.067 | 0.013 | 0.417 | 0.179 | 0.104 |
| Filosa-Imbricatea | 0.000 | 0.217 | 0.000 | 0.003 | 0.000 | 0.170 | 0.000 | 0.006 | 0.000 |
| Filosa-Thecofilosea | 0.523 | 0.253 | 0.054 | 0.136 | 0.200 | 0.182 | 0.051 | 0.087 | 0.141 |
| Bacillariophyta | 28.366 | 65.845 | 28.657 | 13.502 | 10.267 | 60.215 | 21.836 | 7.137 | 87.741 |
| Bolidophyceae-and-relatives | 0.131 | 1.419 | 0.531 | 0.000 | 0.467 | 1.734 | 0.647 | 0.066 | 0.062 |
| Chrysophyceae-Synurophyceae | 0.523 | 0.530 | 0.000 | 0.363 | 0.000 | 1.158 | 0.096 | 0.021 | 0.010 |
| Dictyochophyceae | 0.131 | 0.128 | 0.009 | 0.036 | 0.133 | 0.030 | 0.058 | 3.775 | 0.032 |
| Pelagophyceae | 0.915 | 9.536 | 0.459 | 4.601 | 3.267 | 11.813 | 1.899 | 0.370 | 0.180 |
| Phaeophyceae | 9.542 | 1.447 | 0.480 | 3.597 | 3.867 | 0.653 | 22.062 | 11.363 | 0.228 |
| Xanthophyceae | 0.000 | 0.000 | 0.000 | 0.000 | 0.000 | 0.000 | 0.130 | 0.000 | 0.000 |
| MAST | 0.000 | 1.160 | 0.157 | 1.137 | 0.467 | 1.056 | 0.626 | 0.099 | 0.115 |
| **Order** |  |  |  |  |  |  |  |  |  |
| Haptoria | 0.000 | 0.008 | 0.000 | 0.000 | 0.000 | 0.000 | 0.020 | 0.132 | 0.000 |
| Cyrtophoria | 0.110 | 0.010 | 0.037 | 0.000 | 0.043 | 0.000 | 0.000 | 0.036 | 0.000 |
| Suctoria | 0.221 | 0.025 | 0.238 | 0.052 | 0.087 | 0.000 | 0.136 | 0.034 | 0.001 |
| Choreotrichia | 0.110 | 0.124 | 0.000 | 0.161 | 0.043 | 0.145 | 0.122 | 0.031 | 0.004 |
| Oligotrichia | 1.103 | 0.540 | 0.067 | 1.271 | 0.476 | 0.837 | 1.197 | 3.787 | 0.044 |
| unclass. Spirotrichea | 0.221 | 0.060 | 0.012 | 0.327 | 0.346 | 0.350 | 0.122 | 0.375 | 0.041 |
| unclass. Ciliophora | 0.000 | 0.036 | 0.042 | 0.000 | 0.346 | 0.003 | 0.051 | 0.012 | 0.001 |
| Suessiales | 0.000 | 0.059 | 0.000 | 0.184 | 0.000 | 0.000 | 0.045 | 0.000 | 0.001 |
| Dino-Group-I | 28.776 | 4.781 | 54.202 | 36.249 | 34.156 | 2.946 | 30.909 | 5.444 | 0.039 |
| Dino-Group-II | 0.000 | 0.247 | 0.037 | 0.159 | 0.173 | 0.498 | 0.366 | 0.084 | 0.103 |
| unclass. Syndiniales | 0.000 | 0.052 | 0.000 | 0.000 | 0.000 | 0.118 | 0.040 | 0.000 | 0.000 |
| unclass. Dinophyceae | 8.159 | 5.056 | 1.042 | 3.969 | 7.749 | 4.126 | 6.176 | 19.206 | 7.676 |
| Dolichomastigales | 0.331 | 0.184 | 0.012 | 0.054 | 0.087 | 0.007 | 0.199 | 0.001 | 0.223 |
| Mamiellales | 0.000 | 0.188 | 0.000 | 0.006 | 0.000 | 0.161 | 0.091 | 0.001 | 0.001 |
| Chlorellales | 0.331 | 1.422 | 0.000 | 0.438 | 0.000 | 2.203 | 0.309 | 0.014 | 0.002 |
| Ulotrichales | 0.441 | 0.077 | 0.040 | 0.000 | 0.000 | 0.000 | 0.051 | 0.013 | 0.068 |
| Ulvales-relatives | 0.221 | 0.003 | 0.092 | 0.065 | 0.000 | 0.071 | 0.102 | 0.019 | 0.000 |
| Corallinales | 3.087 | 0.031 | 0.000 | 0.115 | 0.000 | 0.000 | 0.091 | 0.044 | 0.030 |
| Gigartinales | 0.000 | 0.022 | 0.000 | 0.000 | 0.043 | 0.087 | 0.235 | 0.040 | 0.076 |
| unclass. Florideophyceae | 0.000 | 0.016 | 0.040 | 0.101 | 0.000 | 0.000 | 0.105 | 0.041 | 0.017 |
| Kinetoplastida | 0.000 | 0.004 | 0.000 | 0.000 | 0.000 | 0.000 | 0.037 | 0.115 | 0.006 |
| unclass. Cryptophyceae | 4.961 | 1.877 | 1.034 | 6.175 | 7.922 | 5.078 | 1.319 | 34.316 | 1.380 |
| Phaeocystales | 0.000 | 0.000 | 0.000 | 0.198 | 0.000 | 0.000 | 0.000 | 0.000 | 0.000 |
| unclass. Telonemia | 0.110 | 0.216 | 0.015 | 0.210 | 0.087 | 0.434 | 0.216 | 0.083 | 0.141 |
| Saccharomycotina | 0.000 | 0.017 | 0.184 | 0.000 | 0.000 | 0.020 | 0.250 | 0.097 | 0.000 |
| Ustilaginomycotina | 0.000 | 0.226 | 0.025 | 0.410 | 0.130 | 0.061 | 0.221 | 0.032 | 0.011 |
| unclass. Hacrobia | 2.205 | 0.017 | 0.020 | 0.182 | 0.043 | 0.010 | 0.346 | 0.150 | 0.096 |
| Marimonadida | 0.000 | 0.181 | 0.000 | 0.002 | 0.000 | 0.135 | 0.000 | 0.000 | 0.000 |
| Cryomonadida | 0.441 | 0.211 | 0.045 | 0.091 | 0.130 | 0.145 | 0.043 | 0.073 | 0.131 |
| unclass. Bacillariophyta | 23.925 | 54.845 | 23.562 | 9.006 | 6.667 | 47.757 | 18.101 | 6.002 | 81.407 |
| unclass. Bolidophyceae-and-relatives | 0.110 | 1.182 | 0.437 | 0.000 | 0.303 | 1.375 | 0.536 | 0.055 | 0.057 |
| unclass. Chrysophyceae-Synurophyceae | 0.441 | 0.442 | 0.000 | 0.242 | 0.000 | 0.918 | 0.079 | 0.018 | 0.010 |
| unclass. Dictyochophyceae | 0.110 | 0.107 | 0.007 | 0.024 | 0.087 | 0.024 | 0.048 | 3.175 | 0.030 |
| unclass. Pelagophyceae | 0.772 | 7.943 | 0.377 | 3.069 | 2.121 | 9.369 | 1.574 | 0.311 | 0.167 |
| unclass. Phaeophyceae | 8.049 | 1.205 | 0.394 | 2.399 | 2.511 | 0.518 | 18.288 | 9.557 | 0.212 |
| unclass. Xanthophyceae | 0.000 | 0.000 | 0.000 | 0.000 | 0.000 | 0.000 | 0.108 | 0.000 | 0.000 |
| MAST-1 | 0.000 | 0.219 | 0.012 | 0.000 | 0.130 | 0.111 | 0.159 | 0.017 | 0.060 |
| MAST-7 | 0.000 | 0.454 | 0.000 | 0.262 | 0.000 | 0.454 | 0.071 | 0.004 | 0.001 |
| MAST-9 | 0.000 | 0.231 | 0.072 | 0.000 | 0.000 | 0.219 | 0.250 | 0.011 | 0.000 |
| unclass. MAST | 0.000 | 0.023 | 0.045 | 0.496 | 0.173 | 0.000 | 0.040 | 0.008 | 0.025 |
| unclass. Stramenopiles | 0.110 | 0.648 | 0.062 | 0.660 | 1.039 | 0.921 | 0.460 | 0.553 | 0.641 |
| unclass. Eukaryote | 15.656 | 16.706 | 17.777 | 33.299 | 35.065 | 20.689 | 17.105 | 15.891 | 7.219 |

For Class and Order, only taxa above 0.1% are indicated. Classification is according to PR2 database.
